# Single‐Cell Transcriptomics Reveals the Potential Role of GZMK+ CD8+ T Cells in Cell Senescence of Triple‐Negative Breast Cancer

**DOI:** 10.1155/ijog/4215646

**Published:** 2026-04-17

**Authors:** Dan Zhou, Tianhui Hu, Beibei Xu, Jiachen Zhu, Miaomiao Ma, Wenqing Zhang, Liangxi Xie, Zhong Ouyang, Yidan Xu, Yongcheng Su

**Affiliations:** ^1^ Fujian-Taiwan Smart Health and Elderly Care Research Center, Xiamen City University, Xiamen, 361008, China; ^2^ Department of Breast Surgery, The First Hospital of Xiamen University, School of Medicine, Xiamen University, Xiamen, 361004, China, xmu.edu.cn; ^3^ Xiamen Key Laboratory for Tumor Metastasis, Cancer Research Center, School of Medicine, Xiamen University, Xiamen, 361102, China, xmu.edu.cn; ^4^ Shenzhen Research Institute of Xiamen University, Shenzhen, 518057, Guangdong, China, xmu.edu.cn; ^5^ Institute of Synthetic Biology, Shenzhen Institute of Advanced Technology, Chinese Academy of Sciences, Shenzhen, 518055, Guangdong, China, cas.cn; ^6^ Department of Radiation Oncology, Xiang’an Hospital of Xiamen University, Cancer Research Center, School of Medicine, Xiamen University, Xiang’an, Xiamen, 361100, China, xmu.edu.cn; ^7^ Department of Public Health and Medical Technology, Xiamen Medical College, Xiamen, 361023, China, xmmc.com.cn

**Keywords:** cell senescence, Mendelian randomization, single-cell RNA sequencing, triple-negative breast cancer, tumor microenvironment

## Abstract

Senescence is a critical risk factor for the development of triple‐negative breast cancer (TNBC), yet its specific impact on the disease remains inadequately understood. This study aims to elucidate the role of pivotal genes in mediating the effects of gene expression on cell senescence and TNBC progression. We employed a combination of Mendelian randomization (MR) and single‐cell RNA sequencing to investigate these relationships. A two‐sample MR approach was utilized to assess causal links between pivotal genes and TNBC. Single‐cell transcriptomic analysis of senescence and TNBC datasets from the Gene Expression Omnibus database revealed a significant association between the gene GZMK and increased TNBC risk. Our single‐cell analysis uncovered a complex network of ligand–receptor interactions in GZMK + central memory CD8+ T cells (CD8_CM), which frequently interacted with macrophages, monocytes, and epithelial cells. GZMK expression was correlated with pseudotime progression, indicating its potential role in TNBC development. Enrichment analysis suggested that GZMK + CD8_CM cells play a role in enhancing immune process regulation, pointing to a broader immune response. Bulk sequencing further confirmed the presence of GZMK expression in TNBC samples. Functional experiments demonstrated that GZMK significantly influences the proliferation, migration, and invasion of TNBC cell lines *in vitro*. Our findings reveal intricate interactions among immune cell composition, GZMK expression, senescence, and TNBC, highlighting GZMK as a potential molecular target for therapeutic intervention. This study advances the understanding of TNBC pathogenesis and opens new avenues for precision medicine in TNBC treatment.

## 1. Introduction

Breast carcinoma is the most prevalent malignancy affecting women globally and is the leading cause of cancer‐related deaths in this population [1]. Within the various subtypes of breast neoplasms, triple‐negative breast cancer (TNBC) distinguishes itself as a particularly insidious subtype [2, 3]. It is defined by the lack of expression of the human epidermal growth factor receptor 2, estrogen receptor, and progesterone receptor [4]. TNBC is notoriously resistant to conventional chemotherapeutic regimens, predisposes patients to distant metastatic dissemination, and carries a poor prognosis for those affected [5–9]. The increased likelihood of mutations in the breast cancer susceptibility gene 1 among TNBC cohorts further exacerbates the aggressiveness of this disease variant [10]. The confluence of these factors contributes to the poor prognosis for patients with TNBC, underscoring the urgent need to develop therapeutic modalities that extend beyond conventional chemotherapy [9, 11].

Cellular senescence is a major risk factor for oncogenesis. The interplay between senescence and malignancy has been rigorously investigated through the lens of epigenetics, revealing a complex set of epigenetic changes that suggest a molecular nexus linking these phenomena [12]. The fundamental characteristics of cell senescence include genomic instability, epigenetic alterations, persistent inflammation, ecological imbalance [13], mirror oncogenic catalysts of genomic aberrations, epigenetic reprogramming beyond mutational events, inflammatory processes, and microbiome diversity [14]. Both cell senescence and cancer can be considered meta‐markers of organismal decline [15], with particular emphasis on the inflammatory environment that develops with advancing age. This age‐related inflammatory surge leads to various senescence‐associated pathologies, including osteoarthritis, atherosclerosis, myasthenia gravis, and neuroinflammation [16–18]. However, it concurrently promotes oncogenesis [14, 19]. Within the tumor microenvironment (TME), inflammatory cells secrete numerous factors that foster tumorigenicity [15]. These factors induce genetic instability, stimulate cell growth, encourage blood vessel formation, disrupt metabolic control, alter the structure of the extracellular matrix, facilitate cancer cell invasion and spread, maintain cancer stem cells, and evade immune system monitoring [20–23]. Age has been substantiated as a harbinger of diminished survival prospects and an independent prognostic indicator of adverse outcomes in breast cancer [24]. Vaena et al. demonstrated that senescence and ceramide‐dependent mitochondrial autophagy weaken the antitumor function of T cells [25], promoting mouse and human hepatocellular carcinoma (HCC) growth and worsening the survival of human patients with HCC [26]. However, the specific ramifications of senescence in TNBC pathogenesis remain unclear.

Mendelian randomization (MR) has emerged as a robust analytical methodology for discerning causal linkages, susceptible exposures or risk factors, and significant clinical outcomes [27]. This methodology, used to repurpose medications [28] and uncover novel therapeutic targets, demonstrates considerable promise by integrating disease‐specific genome‐wide association studies (GWAS) and expression quantitative trait loci (eQTL) data [29–31]. Our investigation aimed to delineate the potential molecular intermediaries bridging the nexus between cell senescence and TNBC. Subsequent colocalization analysis was conducted to ensure the integrity of the expression of the instrumental variable (IV). Our analysis aimed to unveil the causal gene–TNBC relationship and the mechanisms implicated in TNBC pathogenesis.

## 2. Methods

### 2.1. Data Acquisition

The datasets GSE157007 and GSE161529 were obtained from the Gene Expression Omnibus (GEO) repository. We selected a cohort that included six older samples (F020, F021, F023, OH14, OH15, and OH17), three young adult specimens (F012, F013, and F014), four individuals with TNBC (GSM4909281, GSM4909282, GSM4909283, and GSM4909284), and three normal control samples (GSM4909253, GSM4909254, and GSM4909257) for 10X single‐cell sequencing endeavors. Complementary cohorts (GSE38959, GSE45827, and GSE65194) were used for bulk RNA analyses to enrich our findings. To elucidate the potential causal relationship between the DEGs and TNBC, we performed bidirectional MR on independent datasets (ieu‐a‐1128 and ieu‐a‐1135). An initial hypothesis positioned DEGs as potential risk factors for breast cancer, and subsequently, the inverse relationship was examined. Using a bifurcated MR methodology, we examined the directive capacity of pivotal genes in breast cancer pathology. The genetic instruments employed were single‐nucleotide polymorphisms (SNPs) selected from an extensive European GWAS compendium accessible at https://gwas.mrcieu.ac/. The single‐cell RNA‐seq cohorts analyzed in this study include six older samples, three younger adult samples, four TNBC samples, and three normal control samples. Limited information on TNBC staging and incomplete clinical annotations across these datasets constrain our ability to perform systematic stratified analyses by age or tumor stage. We acknowledge this limitation and suggest that future work incorporate datasets with richer clinical metadata or harmonize across additional sources to enable more granular age‐ and stage‐specific immune microenvironment evaluations.

### 2.2. Statistical Analyses of Single‐Cell RNA Sequencing Data

This study used data from various samples, including normal controls, geriatric patients, and patients with TNBC. Seurat objects were created for individual samples before being synthesized into an integrated Seurat entity for uniform analysis [32]. Cells were excluded based on the following criteria: mitochondrial content exceeding 10% and a unique gene count below 200 or above 4000. For dimensionality reduction and clustering, a subset of 2000 genes exhibiting the greatest variability in expression was selected for principal component analysis (PCA). The Harmony algorithm was used to reduce batch biases, and Uniform Manifold Approximation and Projection (UMAP) technology was employed to graphically represent the data in two dimensions, facilitating the unsupervised identification of cellular subsets. Annotation of cellular subpopulations was performed using the SingleR platform [33], with key marker genes visualized through the DimPlot and FeaturePlot modalities. For functional annotation of the differential genes, a twofold change and a modified *p* < 0.05 were necessary. The resulting differentially expressed genes (DEGs) were visualized as a heatmap for the gene expression analysis. To investigate temporal sequences or developmental arcs, Monocle2 [34] and Slingshot [35] were used to analyze developmental trajectories, with trajectories subsequently depicted using UMAP. For Slingshot, pivotal genes driving the trajectory were identified using expedited logistic regression functions such as find_switch_logistic_fastglm. These genes are believed to play a crucial role in trajectory evolution, with gene expression trends across pseudotime illustrated using plot_timeline_ggplot. During metabolic interrogation, the scMetabolism tool [36] was used to assess the metabolic flux of different cellular entities. Additionally, cellular interaction analysis was performed using tools such as CellChat [37] and CellPhoneDB [38], which involved delineating ligand–receptor pairs and computing the communicative propensities among various cellular clusters. Expression thresholds were applied to improve network robustness. For each cell group, a gene’s expression is considered only if at least 25% of cells express it; otherwise, the gene’s mean expression in that group is set to zero. A minimum participating‐cell threshold of 10 was used, and ligand–receptor pairs involving fewer than 10 participating cells were excluded from the analysis. These thresholds were applied during network inference and significance testing, and only interactions meeting these criteria are reported. A supporting table lists all examined ligand–receptor pairs with per‐group expression, threshold status, and whether they passed the criteria. pySCENIC software was used for TF regulatory network analysis [39].

### 2.3. CD8_CM Core Marker Gene eQTL and TNBC MR Inquiry

The gene expression dataset underwent preprocessing, encompassing normalization, batch disparity alleviation, and handling of missing data. Identifying key marker genes specific to CD8_CM involved comparing them with other T cell and cellular populations. We explored eQTLs related to the hallmark gene of CD8_CM. Gene nomenclature was converted to ENSEMBL identifiers for data consistency. Subpar SNPs were filtered out, and genotypic data were calibrated. Stringent significance criteria for eQTLs were set at a *p*‐value of 5  ×  10^−8^. SNPs pertinent to the key marker gene were selected from the “ieu‐a‐1128” GWAS dataset to serve as instruments for the MR analysis. Candidate SNPs meeting the rigorous requirements based on *R* ^2^ and *F*‐statistics were retained. MR analysis was performed using the TwoSampleMR framework to explore the associations with these IVs [40]. This involved creating a “harmonized_dat” array by integrating outcome and IV data. Bidirectional MR analysis followed this step. Gene expression eQTL data were processed using the vcfR toolkit [41].

### 2.4. Colocalization Analysis, Regional Association Plot, and PhenoScanner Analysis

We performed a colocalization analysis using COLOC [42]. This involved analyzing the genotype data in VCF format and associated information to gather eQTL details for specific genes. Subsequently, we selected eQTLs within a specified region to generate a regional association plot. Using the locuscomparer package [43], we visualized eQTL and GWAS association information, providing a clear graphical representation for further analysis. We then performed the analysis using PhenoScanner [44]. Additionally, we employed the Steiger directionality test to examine the causal relationship between postulated exposure and outcomes [45]. This approach helped verify the placement of SNPs in the causal pathway. We performed MR‐PRESSO to evaluate horizontal pleiotropy among the SNPs used as IVs for GZMK. The global test, outlier test, and distortion test were applied to assess potential pleiotropy and its impact on the causal estimate. As the global test indicated no evidence of pleiotropy (*p* > 0.05) and no outlier SNPs or distortion effects were detected, the pleiotropy assumption is satisfied in our data.

### 2.5. Cell Lines, Cell Culture, and Cell Transfection

The MDA‐MB‐231 and MDA‐MB‐453 cell lines were obtained from the Cell Bank of the Chinese Academy of Sciences (Shanghai, China) and authenticated for scientific use. Cells were cultured in a humidified incubator with 5% CO_2_ at 37°C, using DMEM and RPMI 1640 medium, respectively. Each medium was supplemented with 10% fetal bovine serum (HyClone, Logan, UT, USA) and antibiotics, including 100 U/mL penicillin and 100 μg/mL streptomycin (Life Technologies, Carlsbad, CA, USA). A lentiviral expression system was employed for knockdown experiments to deliver short hairpin RNA (shRNA) targeting GZMK. Briefly, lentiviral vectors (plv3‐shGZMK/shCtrl), in conjunction with PHR and VSVG plasmids, were transfected into HEK293T cells using polyethyleneimine (Invitrogen, Thermo Fisher Scientific, Shanghai, China). After 48 h in culture, the viral supernatant was harvested, centrifuged, and collected. Subsequently, MDA‐MB‐231 and MDA‐MB‐453 cells were transduced with this viral supernatant at a concentration of 2 μg/mL, followed by selection with 1 μg/mL puromycin (Invitrogen, Thermo Fisher Scientific, Shanghai, China). Effective knockdown of the target protein was confirmed via Western blotting. The shRNA sequences employed were as follows: GAG​GGC​CTA​TTT​CCC​ATG​A (shCtrl), GGA​AAG​AAG​TGT​CAC​CTC​ATT​CAA​GAG​ATG​AGG​TGA​CAC​TTC​TTT​CC (shGZMK‐1), and GAT​CCT​CAA​TCA​AAT​GAT​ATT​CAA​GAG​ATA​TCA​TTT​GAT​TGA​GGA​TC (shGZMK‐2).

### 2.6. Cell Proliferation Analysis

Cell proliferation was assessed using the methylthiazole tetrazolium (MTT) assay according to the manufacturer’s instructions. Stable cell lines expressing shRNA were seeded into 96‐well plates at a density of 2500 cells per well, with five replicates per condition. Postadherence, the culture medium was replaced with 100 μL of fresh medium containing 20 μL of MTT reagent, and cells were incubated for 4 h to enable the formation of formazan crystals. Absorbance was measured at 490 nm, 15 min after the addition of 150 μL of dimethyl sulfoxide for solubilization. Proliferation was measured at 24, 48, 72, and 96 h, with optical density (OD) values normalized against the initial OD of the first 96‐well plate.

In accordance with established protocols, cell proliferation was also evaluated via colony formation assays and EdU incorporation tests [46]. For the colony formation assay, cells were plated in 6‐well plates at a density of 500 cells per well and allowed to grow for 2 weeks. The medium was removed from the wells, and the cells were rinsed once with phosphate‐buffered saline (PBS) and then fixed with 4% paraformaldehyde for 15 min. After washing three times with PBS, the cells were stained with 0.5% crystal violet for 30 min, followed by additional washes to remove excess stain. Colonies were subsequently quantified. For the EdU assay, MDA‐MB‐231 and MDA‐MB‐453 cells were seeded at a density of 8 × 10^3 cells per well in 96‐well plates. EdU incorporation was measured using the Cell‐Light EdU Apollo 567 *In Vitro* Kit (RiboBio, Guangzhou, China), according to the manufacturer’s protocols. Fluorescent images of Cy3‐EdU and Hoechst‐stained cells were captured using an Olympus IX73 confocal microscope at 40× magnification.

### 2.7. Wound Healing Assay

The wound healing assay was performed following established protocols [46]. For spatial orientation during imaging, horizontal and vertical reference lines were marked on the bottom of 6‐well plates. Cells transduced with shCtrl and shGZMK constructs were seeded (approximately 5 × 10^5^ cells per well) and incubated for 12 h in a serum‐free medium. Linear scratches were made across the cell monolayer using the tip of a 10 μL pipette. Subsequently, the cells were washed gently with PBS and replaced with fresh, serum‐free medium. Images of the wound area were captured at 0‐, 24‐, and 48‐h time points using a 10 × objective. The wound healing rate was quantified using ImageJ software (Version 1.46).

### 2.8. Migration and Invasion Assays

Transwell migration assays were conducted using Transwell chambers (Corning, NY, USA). Approximately 20,000 cells resuspended in serum‐free medium were seeded into the upper chamber, while the lower chamber contained medium supplemented with serum. For invasion assays, Matrigel (Corning, NY, USA) was added to 24‐well plates. Following a 24‐h incubation at 37°C in 5% CO_2_, noninvasive cells on the upper side of the membrane were removed using cotton swabs. The invaded cells on the lower side of the membrane were fixed, stained with 0.5% crystal violet, and counted under an Olympus IX73 microscope.

### 2.9. Western Blotting

Western blotting was conducted according to established methodologies [47]. Proteins were extracted using RIPA buffer and quantified using the bicinchoninic acid protein quantification kit (Thermo Fisher Scientific, Cat. No. 23228). Protein lysates (15–30 μg) were separated using 10% sodium dodecyl sulfate–polyacrylamide gel electrophoresis and transferred to polyvinylidene fluoride membranes (Merck Millipore Ltd, Darmstadt, Germany). Membranes were blocked with 10% nonfat dry milk. Immunoblotting was performed using anti‐GAPDH (internal control, 1:10,000; Sigma, USA), anti‐p‐p38 MAPK (Cat No.YP0338; ImmunoWay), anti‐p38 MAPK (Cat No.YT6121; ImmunoWay), anti‐p‐ERK (Cat No. 9106S; CST), anti‐ERK (Cat No. 9102S; CST), and anti‐GZMK (target protein, 1:500; Abcam, China) antibodies.

### 2.10. Data Analysis

The statistical tests were performed using R Version 4.1.1, with a significance level set at *p* < 0.05. This stringent criterion ensured that only findings with a high probability of causal relationships were considered, bolstering the reliability and validity of the investigational outcomes.

## 3. Results

### 3.1. Single‐Cell Transcriptomic Exploration of TNBC, Aging, and Control Groups

In this study, we incorporated six aging, three young, four TNBC cases, and three healthy control samples from the GSE1570075 and GSE151529 datasets. We implemented a 10× single‐cell RNA‐seq analysis, followed by an initial screen to filter out low‐quality data (Figure S1A), resulting in the retention of 121,610 cells for further investigation. The data normalization process is shown in Figure S1B. To mitigate the impact of batch effects among samples, we used the Harmony approach to integrate and standardize the samples and further applied PCA for dimensionality reduction and clustering (Figures S1C and S1D). Subsequently, we used the SingleR software package to redefine 25 clusters, applied the UMAP algorithm, and used the “DimPlot” function to visualize cell distribution (Figure 1(a)), categorizing them based on disease type (Figure 1(b)). Density plots in Figure 1(c) show the expression patterns of marker genes in various cellular phenotypes, such as T cells, epithelial cells, endothelial cells, macrophages, tissue stem cells, B cells, monocytes, natural killer (NK) cells, platelets, and erythroblasts. The cell proportion maps in Figure 1(d) illustrate the distribution and expression of hallmark genes in various cell types. This facilitated a deeper understanding of their functional roles and distinctive characteristics.

FIGURE 1Single‐cell transcriptome data integration to identify the tumor microenvironment (TME) in old and triple‐negative breast cancer (TNBC) samples. (a) The cell types from scRNA‐seq data were displayed using the UMAP projection from old and TNBC samples. (b) The cell types of scRNA‐seq data were displayed using the UMAP projection with different tissue types. (c) Visualization of marker gene expression in each cluster using a density plot through the Nebulosa package. The right side of the plot shows the scale of expression density. (d) The distribution of various cell subgroups derived from different patients. (e) Heat map showing 10 different clusters of differentially expressed genes (DEGs). Red in the heatmap represents high expression, and blue represents low expression. (f) Enrichment analysis of DEGs in the 10 T‐cell subpopulations.

**Figure:**
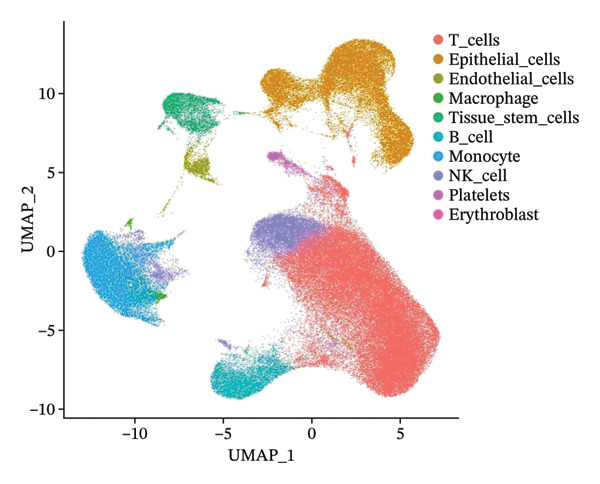
(a)

**Figure:**
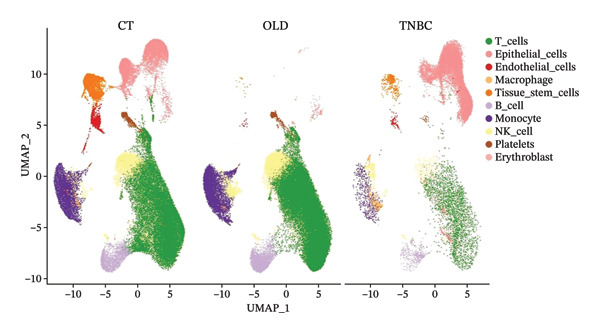
(b)

**Figure:**
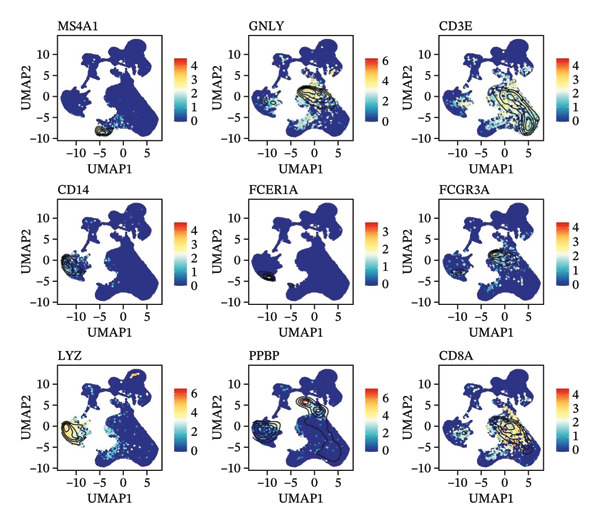
(c)

**Figure:**
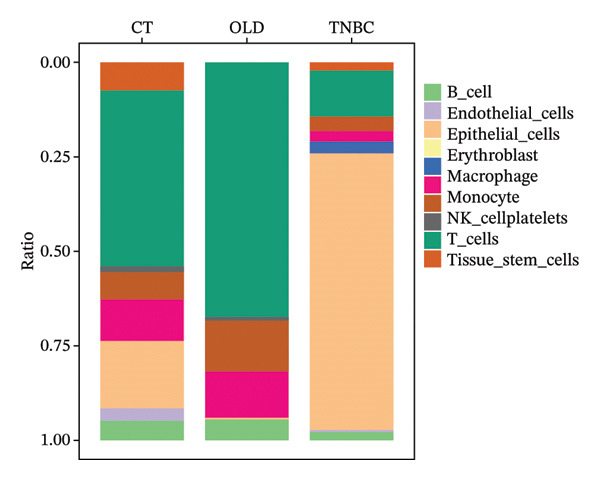
(d)

**Figure:**
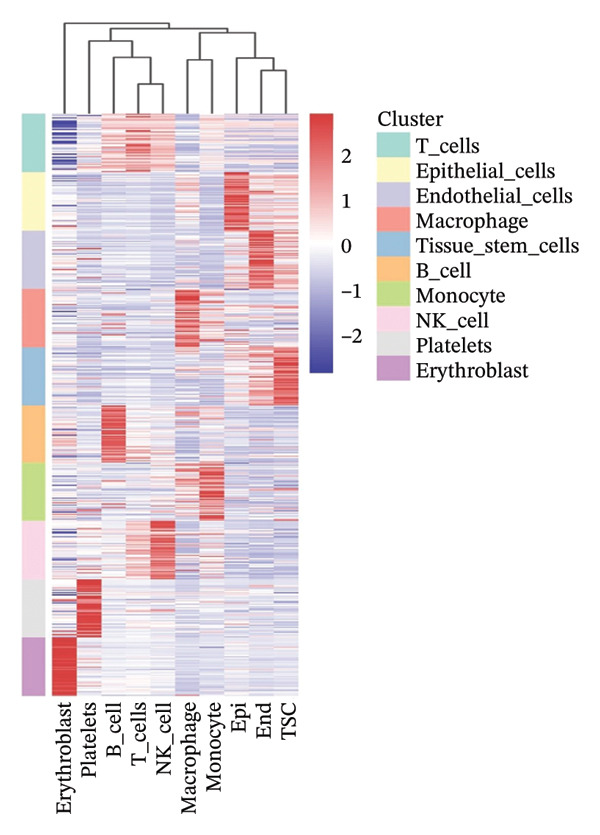
(e)

**Figure:**
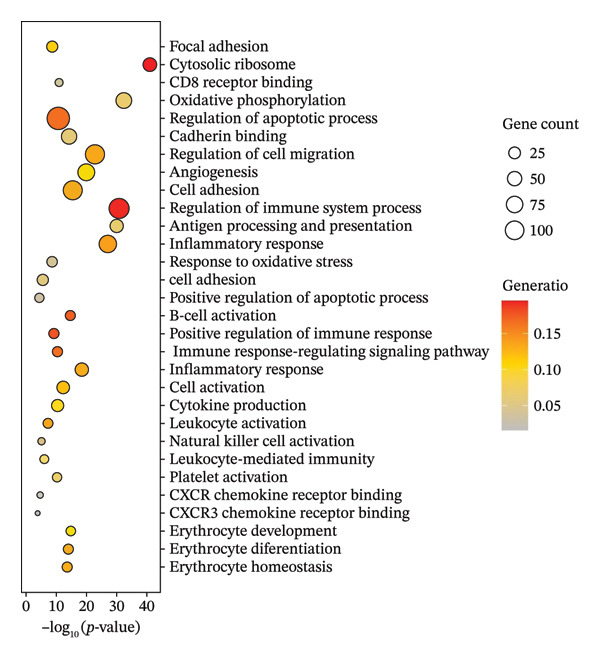
(f)

Regarding the 10 identified cell phenotypes, genes with fold changes of ≥ 2 or ≤ −2 were categorized as DEGs. The top 50 overexpressed genes in T cells, macrophages, endothelial cells, monocytes, epithelial cells, tissue stem cells, B cells, NK cells, platelets, and erythroblasts were visualized using a heatmap (Figure 1(e)). Gene ontology (GO) analysis of cell type‐specific markers was performed using ToppGene [48] to interpret the functions of DEGs in different cell clusters. We selected GO terms representing the functions of each cell type with a *p*‐value < 0.05. As shown in Figure 1(f) and Table S1, GO enrichment analysis indicated that molecular function GO terms, including T‐cell receptor binding (GO:0042608), MHC Class I protein binding (GO:0042288), and cell adhesion molecule binding (GO:0050839), were significantly enriched in DEGs of T cells, while oxidative phosphorylation (GO:0006119), cadherin binding (GO:0045296), and ATP‐dependent protein folding chaperone (GO:0140662) were significantly enriched in epithelial cells. Our results provide preliminary insight into the functionality of each cell type.

### 3.2. Single‐Cell Transcriptomic Analysis of T‐Cell Types

Cells play a pivotal role in the initiation and development of many physiological and pathological processes, including cancer and aging. Immunosurveillance should be avoided for cancers to transition to a clinically discernible state. Several hallmarks of cell senescence facilitate the immune evasion of cancer, including compromised autophagy, cellular senescence, chronic inflammation, and immune cell senescence, particularly in T lymphocytes [15]. Therefore, a deeper understanding of T‐cell function and the interplay between these processes is crucial for developing effective preventive and therapeutic strategies. We began our investigation by separating T‐cell subpopulations from extensive single‐cell RNA sequencing data. Subsequently, we undertook a series of methodological actions, including preprocessing, dimensionality reduction, clustering, and analytical display of the data. The UMAP and DimPlot methods were used to determine the spatial arrangement of the 19 T‐cell groups, as shown in Figure 2(a). Next, the clusters were segmented according to disease type, as illustrated in Figure 2(b). Dot plots (Figure 2(c)) and cell‐scale plots (Figure 2(d)) delineated the distribution and gene expression profiles of the diverse T‐cell subpopulations. The dispersion of five distinct T‐cell subpopulations is shown in Figure 2(e), including naïve CD4 T cells (CD4_Naive), effector memory CD4 T cells (CD4_EM), central memory CD8 T cells (CD8_CM), central memory CD4 T cells (CD4_CM), and CD4 T reg cells (CD4_REG). This dispersion is also segmented based on disease type (Figure 2(f)). The top 50 overexpressed genes of diverse T‐cell subpopulations were visualized using a heatmap (Figure 2(g)). GO analysis was performed to decode the functions of DEGs in divergent cell clusters, as shown in Figure 2(h) and Table S2, revealing that CD8_CM functionally enriched pathways related to CD8 receptor binding, MHC Class II receptor activity, and T cell–mediated cytotoxicity.

FIGURE 2The subtypes of T cells in old and TNBC samples. (a) Feature plot showcasing the distribution of T cells across 18 clusters. (b) Proportion of each cell subset in different samples. (c) Dot plot illustrating the expression of characteristic genes across various cell subsets. (d) Distribution of various T‐cell subgroups derived from different patients. (e) Manually annotated T‐cell subgroups, including CD4_Naive, CD4_REG, CD4_EM, CD4_ CM, and CD8_CM. (f) Distribution of T‐cell subgroups in different samples. (g) Heat map showing five different T‐cell subgroups of DEGs. (h) Enrichment analysis of DEGs in the five T‐cell subpopulations. DEGs: differentially expressed genes; TNBC: triple‐negative breast cancer.

**Figure:**
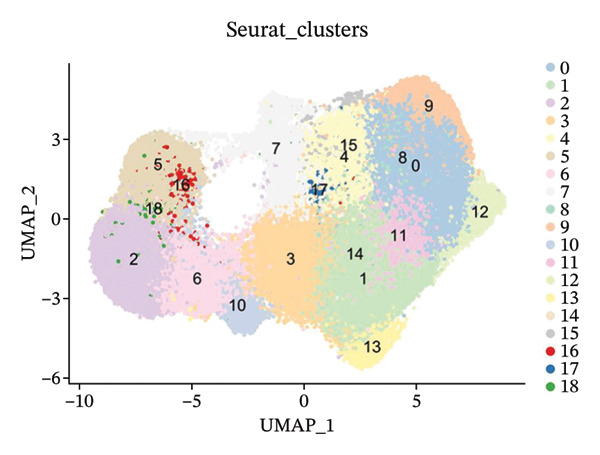
(a)

**Figure:**
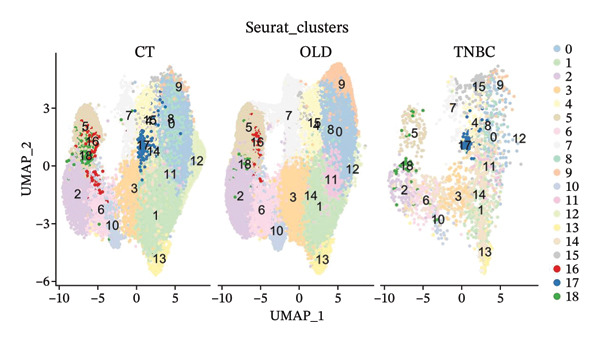
(b)

**Figure:**
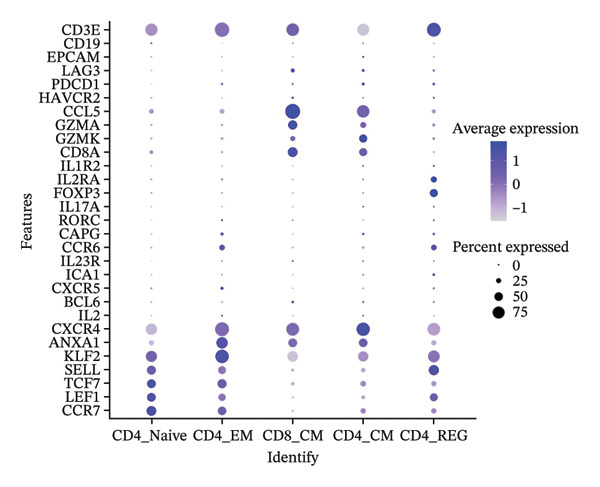
(c)

**Figure:**
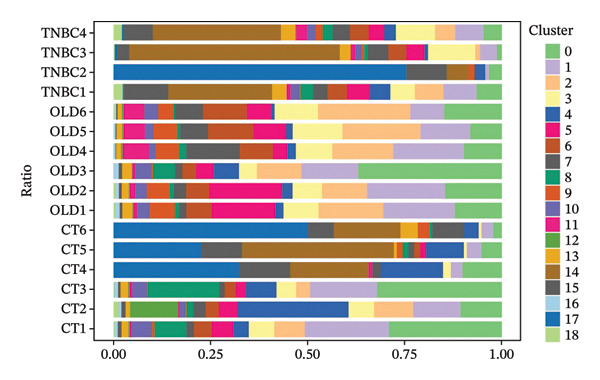
(d)

**Figure:**
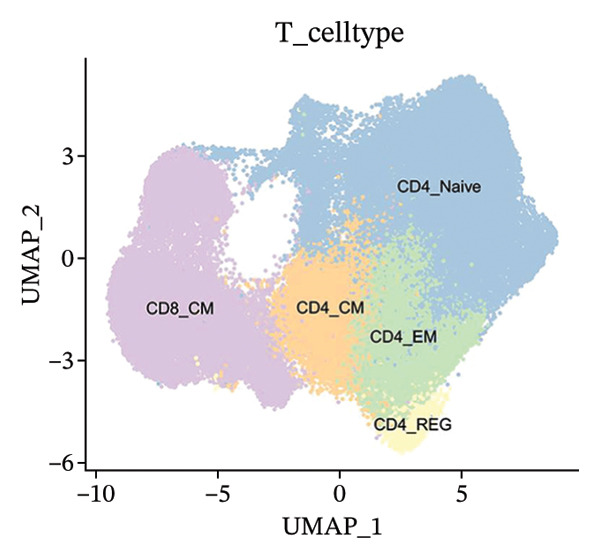
(e)

**Figure:**
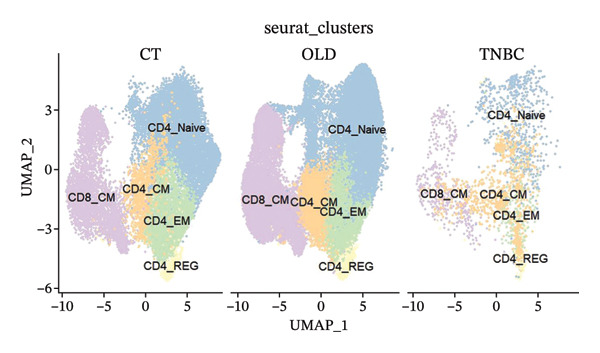
(f)

**Figure:**
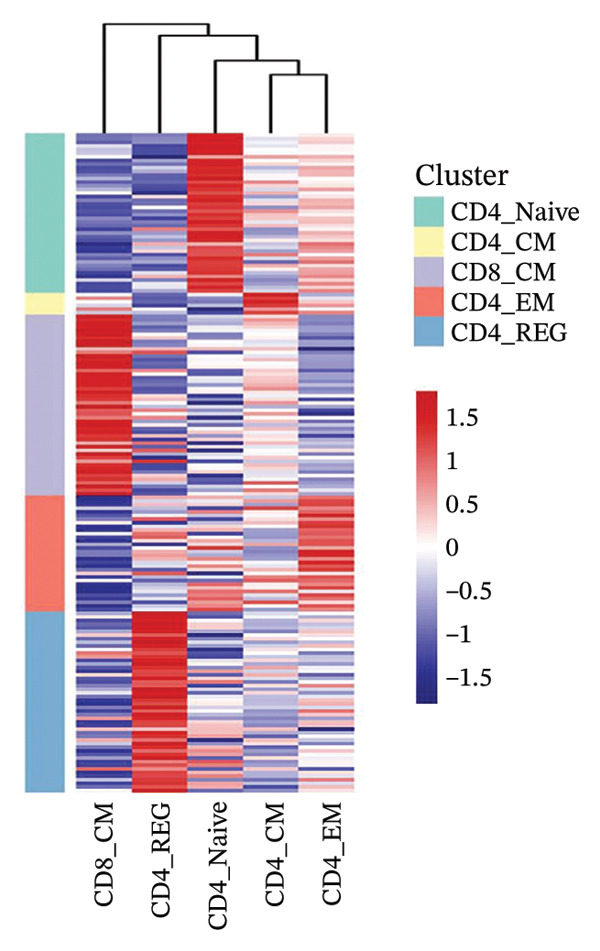
(g)

**Figure:**
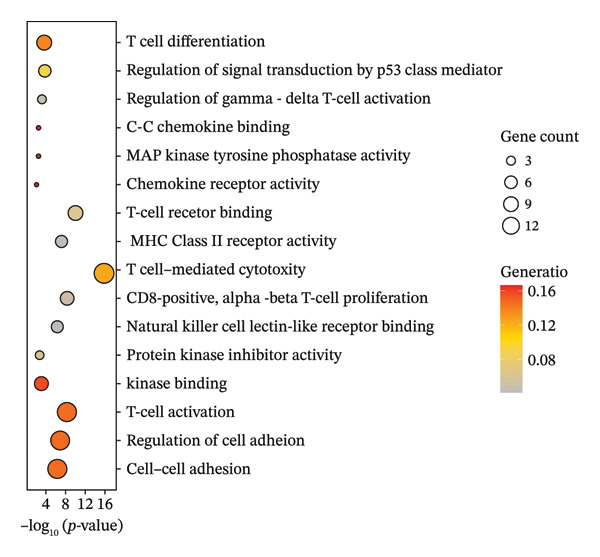
(h)

### 3.3. Analysis of T Cells’ Single‐Cell Trajectory

To investigate the dynamic shift in the composition and transcriptional profile of T cells as the disease progressed, we performed trajectory analysis using Monocle 2. CD4_Naive was identified as the starting point in the trajectory analysis, with CD8_CM ultimately derived from it, suggesting that CD8_CM is a mature form of CD8 T cells (Figures 3(a) and 3(b)). Moreover, based on CytoTRACE, we estimated that CD4_naïve cells had a higher differentiation potential (Figures 3(c) and 3(d)). This finding is consistent with the analysis conducted using the Monocle 2 algorithm. The transcriptional profile changes associated with T‐cell state transitions were analyzed and categorized into three phases (Figure 3(e)). Phase 1 cells exhibited higher expression levels of RPS3A, RPL30, RPL19, RPS12, EEF1B2, and RPS23, whereas Phase 3 cells highly expressed TNFAIP3, BHLHE40, AHNAK, SH3BGRL3, PFN1, CD99, and UB, among other genes. Pathway analysis revealed that Phase 1 cells were involved in T cell–mediated cytotoxicity, regulation of T‐cell activation, and response to tumor cells, whereas Phase 3 cells were involved in cytoplasmic translation, ribosome assembly, and ribosome biogenesis (Figure 3(f) and Table S3).

FIGURE 3Pseudotime analysis of T cells. (a and b) The developmental trajectory of T cells is inferred by Monocle 2. (c and d) CytoTRACE predicts the differentiation status ordering of T‐cell types in old and TNBC samples. (e) Pseudotime heatmap of DEGs reflecting the estimated pseudotime trajectory, hierarchically sorted into three subclusters, and (f) GO enrichment analysis of each subcluster. DEGs: differentially expressed genes; GO, gene ontology; TNBC: triple‐negative breast cancer.

**Figure:**
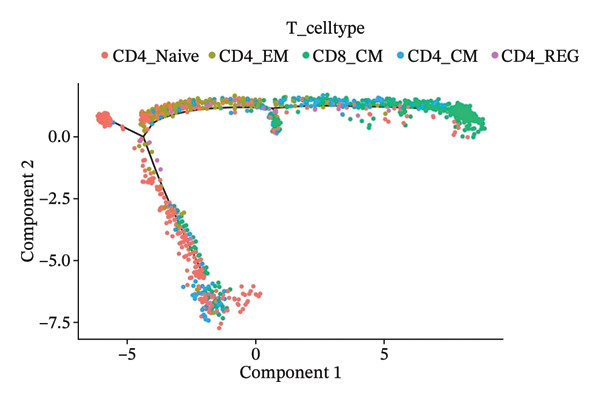
(a)

**Figure:**
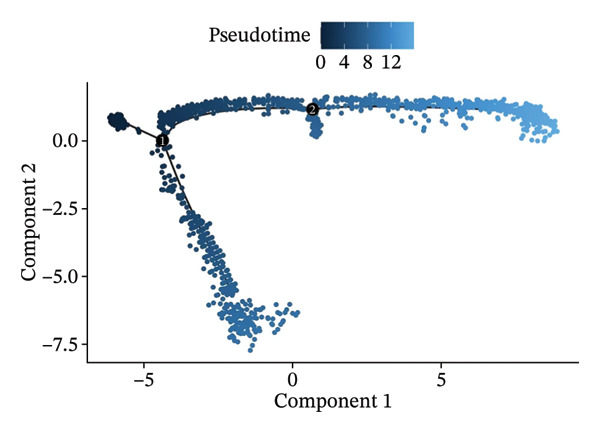
(b)

**Figure:**
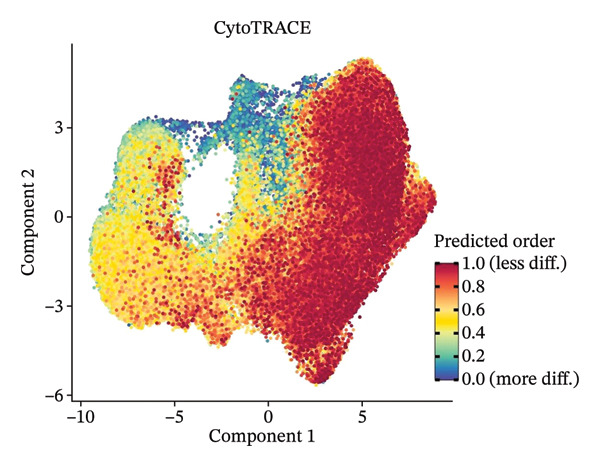
(c)

**Figure:**
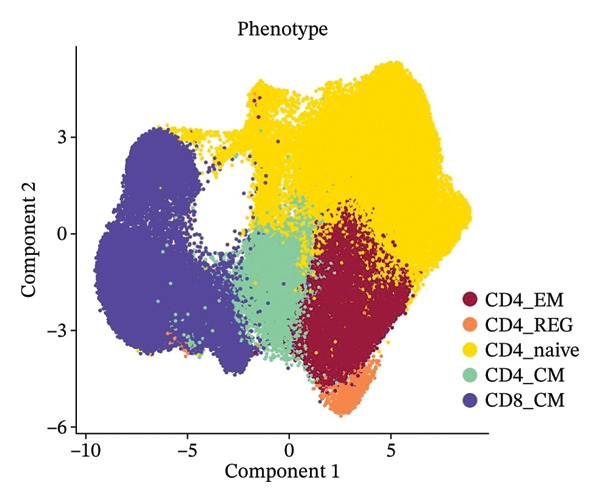
(d)

**Figure:**
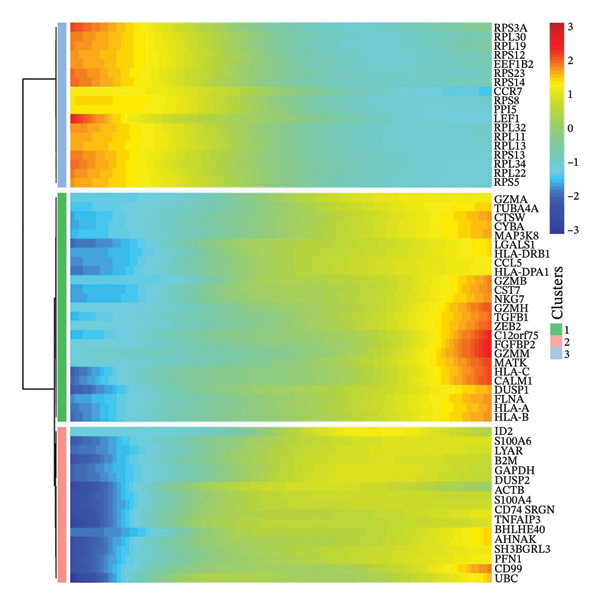
(e)

**Figure:**
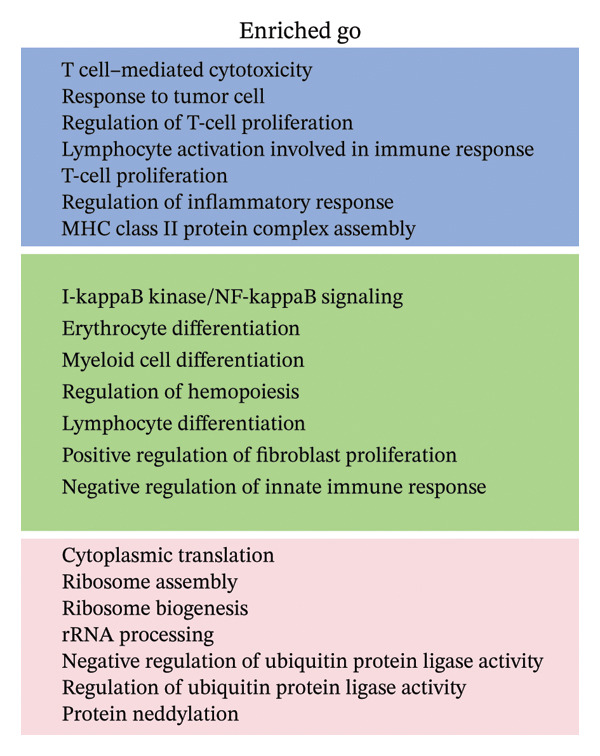
(f)

### 3.4. Cell–Cell Communication Analysis for T‐Cell Types and Transcription Factor (TF) Signatures Predicted by Analysis With pySCENIC

To investigate the function of CD8_CM in aging and TNBC, as well as its relationship with other T‐cell subtypes, we performed a cell communication analysis. We established a communication network between CD8_CM and various T‐cell subsets. Notably, CD8_CM showed significant interactions with endothelial cells, monocytes, and macrophages in TNBC samples (Figure 4(a)), whereas CD8_CM exhibited more frequent communication with NK cells, B cells, monocytes, and CD4_CM in aging samples (Figure 4(b)). Pathway analysis of CD8_CM revealed that MIF‐(CD74+CD44) signaling frequently communicated with B cells, monocytes, and macrophages during aging and TNBC (Figures 4(c) and 4(d)). Therefore, CD8_CM is considered an integral component of both aging and TNBC. To investigate the regulatory programs that cause significant differences in the transcriptional profiles of T‐cell subtypes, we examined the transcriptional regulatory network of T cells. pySCENIC inferred highly activated TFs for each T‐cell subtype (Figure 4(e)). Among these TFs, TBX21 exhibited the highest expression level and transcriptional activity in CD8_CM cells (Figure 4(f)). Additionally, CD4_EM, CD4_REG, CD4_CM, CD4_Naive, NR1D, SIX1, EGF4, and RUNX3 exhibited high expression levels and transcriptional activity (Figures 4(g), 4(h), 4(i), 4(j)). Downstream target genes of TBX21 and NR1D1 included specific marker genes associated with CD8_CM and CD4_EM (Figure 4(k) and Table S4). Therefore, TBX21 and NR1D1 are likely involved in the regulation of CD8_CM and CD4_EM differentiation. These results indicate that TFs, including TBX21 and NR1D, play significant roles in T‐cell subtype differentiation.

FIGURE 4Elucidation of intercellular communication and examination of transcriptional regulons within T cells. (a and b) Architectures of cellular dialog networks delineated for CD8_CM and various cellular entities within the contexts of TNBC (a) and senescence‐associated specimens (b). (c–d) Comparative investigation of enriched signaling pathways between CD8_CM and different cellular cohorts in both TNBC (c) and specimens characterized by Old (d). (e) Heatmap showing the activity of transcription factors in different T‐cell clusters. (f–j) Dot plots showing the transcription factor (TF) regulons that regulate T‐cell subtypes predicted by pySCENIC. UMAP plot, showing the expression of the most active TF in each T‐cell cluster. (f) CD8_CM; (g) CD4_EM; (h) CD4_REG; (i) CD4_CM; (j) CD4_Naive. (k) Analysis map of the TF‐target regulatory network, including TBX21, NR1D1, and their predicted target genes (TGs). TNBC: triple‐negative breast cancer; UMAP: Uniform Manifold Approximation and Projection.

**Figure:**
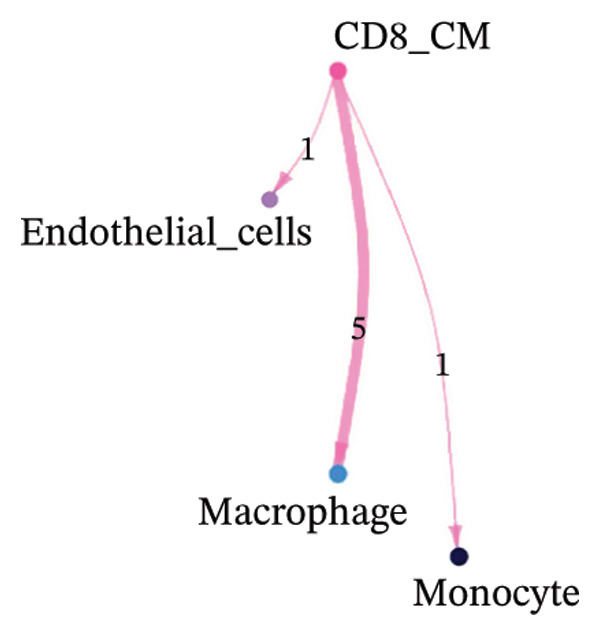
(a)

**Figure:**
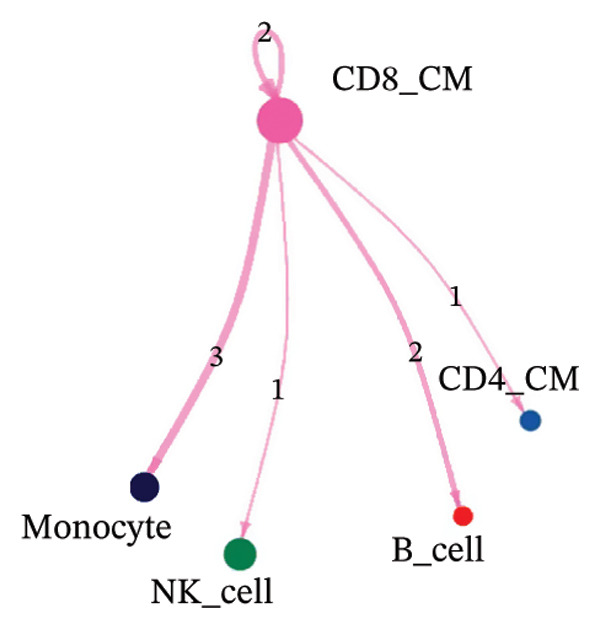
(b)

**Figure:**
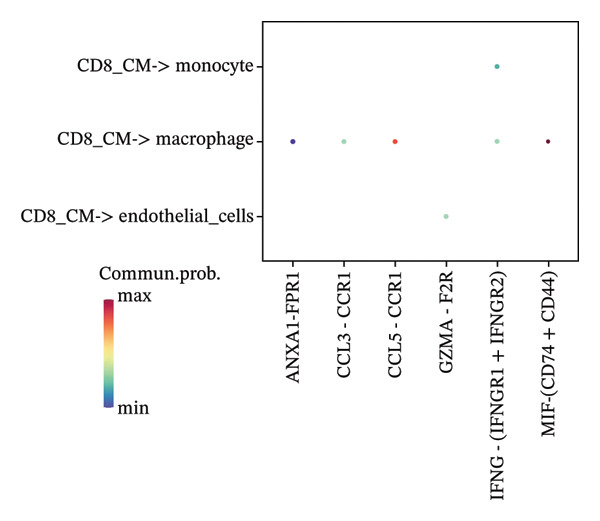
(c)

**Figure:**
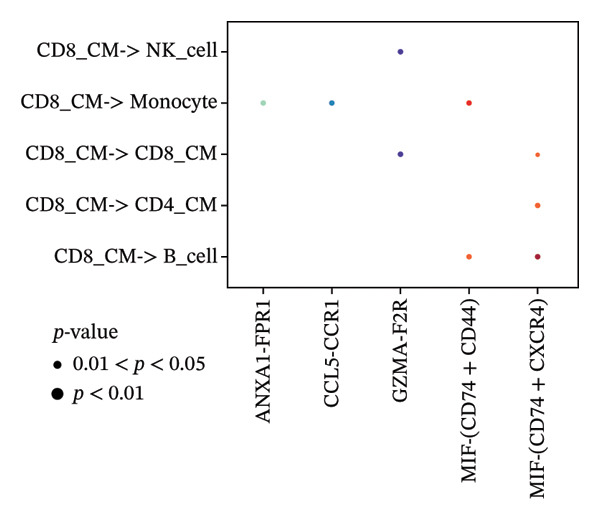
(d)

**Figure:**
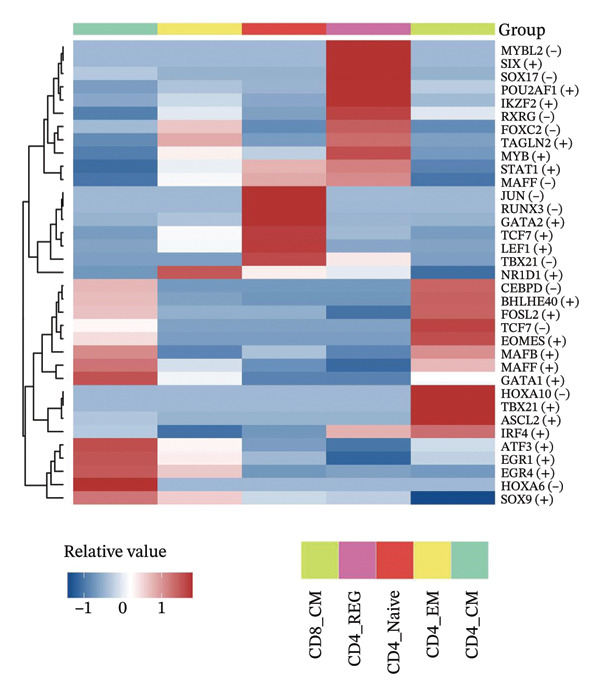
(e)

**Figure:**
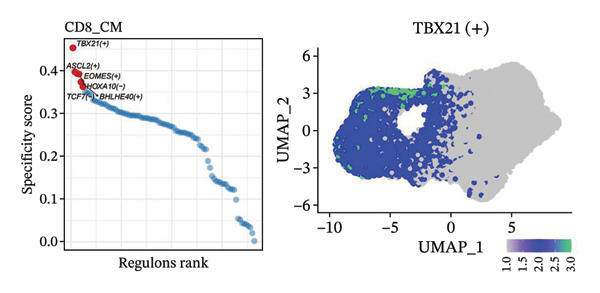
(f)

**Figure:**
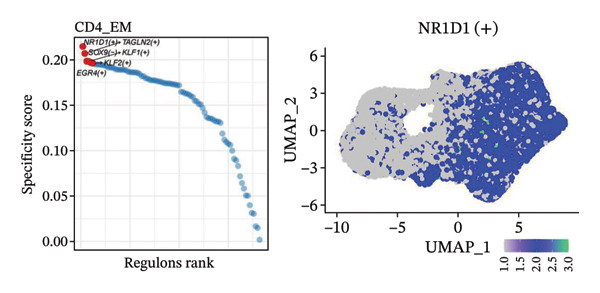
(g)

**Figure:**
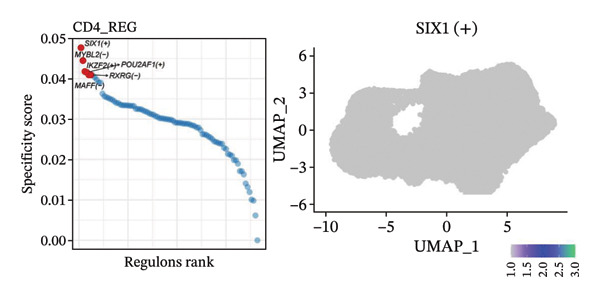
(h)

**Figure:**
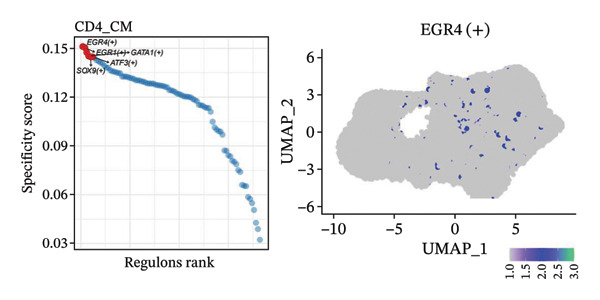
(i)

**Figure:**
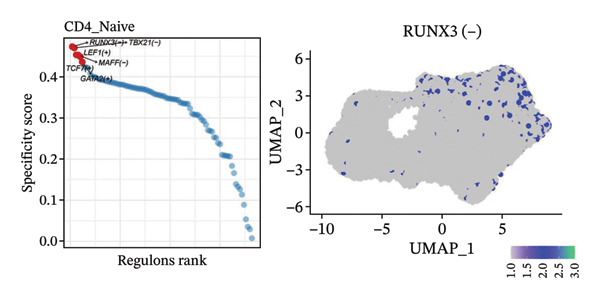
(j)

**Figure:**
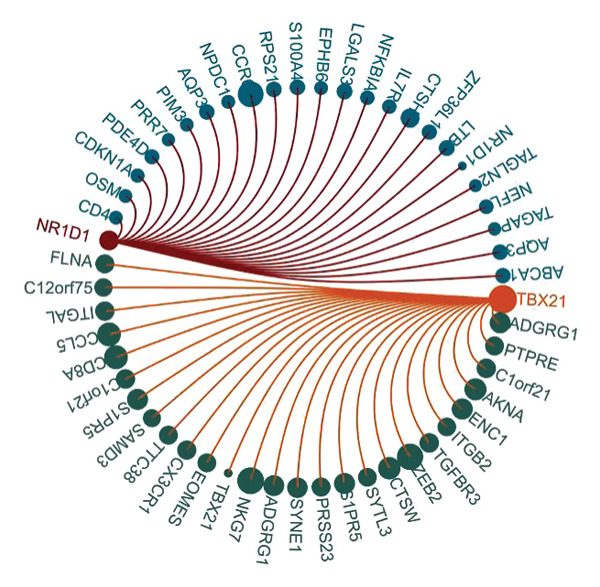
(k)

### 3.5. MR Analysis Identified Four Genes That Were Linked to TNBC

MR analysis was performed on single‐cell RNA sequencing data from senescent cells and TNBC to identify key genes that may influence disease onset and progression. The Seurat tool identified 40 key genes (Table S5) that distinguished CD8_CM from disparate cell subpopulations and T‐cell subsets, serving as essential genes for subsequent analyses. Gene symbols were converted to their respective ENSEMBL identifiers using the org.hs.egg.db package. Two‐sample MR analyses were performed using SNP data associated with key genes for cell senescence and TNBC as exposure variables. The outcome data pertinent to TNBC were extracted from the European Bioinformatics Institute database (ieu‐a‐1128). A volcano plot was created to visually represent these results, clearly displaying genes that were significantly positively and negatively correlated (Figure 5(a)). MR analysis identified four genes significantly associated with risk (Figure 5(b)): *SH2D2A* had an odds ratio (OR) of 1.2285 (95% confidence interval [CI]: 1.0076–1.4978, *p* = 0.04); *GZMK* had an OR of 1.0641 (95% CI: 0.9998–1.1326, *p* = 0.05); *ADGRE5* had an OR of 1.14361 (95% CI: 1.0087–1.2965, *p* = 0.036), and *CD7* had an OR of 1.2197 (95% CI: 1.0317–1.4419, *p* = 0.02). These correlations suggest that certain genetic markers modulate the likelihood of TNBC development.

FIGURE 5Bidirectional MR estimates for the casual associations between key genes and TNBC risk. (a) Forest plot depicting the association of key genes with TNBC risk based on the EBI database (ieu‐a‐1135). (b) Bidirectional Mendelian randomization estimates for the casual associations between key genes and TNBC risk. (c) Funnel plots to visualize the overall heterogeneity of MR estimates for the effect of GZMK on TNBC risk. (d and e) MR scatter plot and a leave‐one‐out plot showing the effect of GZMK on TNBC. (f) Forest plot of MR analysis for nine SNPs of GZMK. EBI: European Bioinformatics Institute; MR: Mendelian randomization; SNP: single‐nucleotide polymorphism; TNBC: triple‐negative breast cancer.

**Figure:**
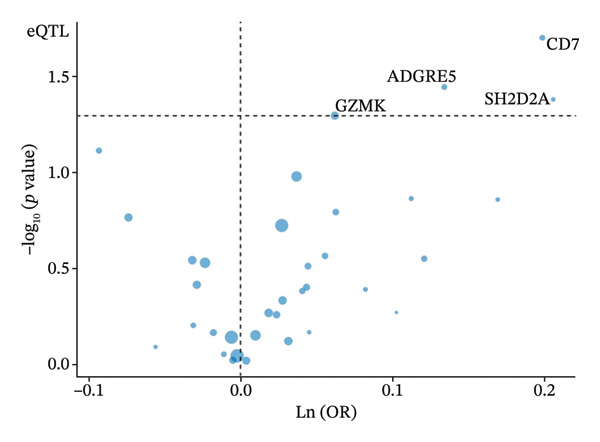
(a)

**Figure:**
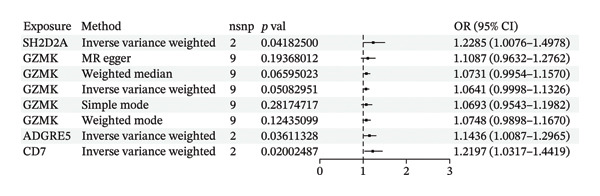
(b)

**Figure:**
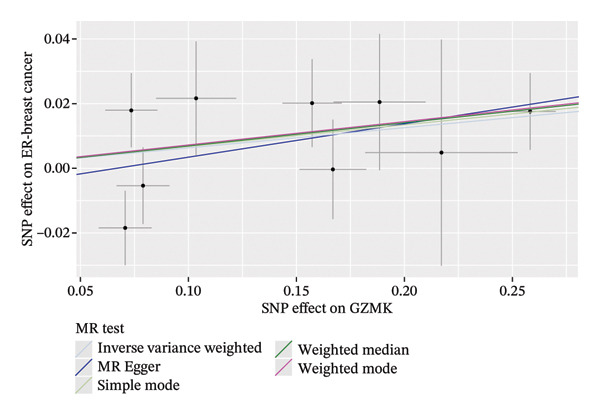
(c)

**Figure:**
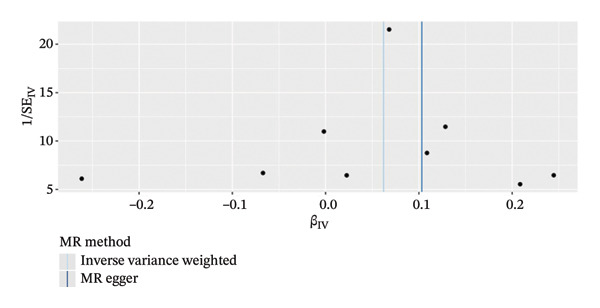
(d)

**Figure:**
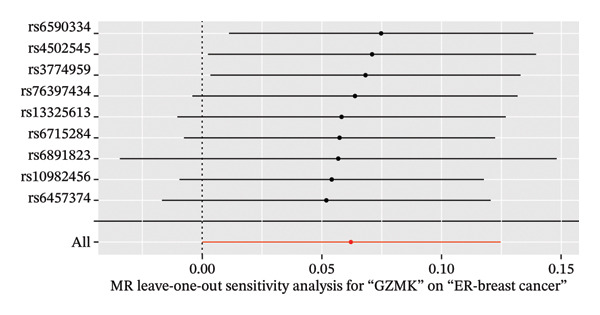
(e)

**Figure:**
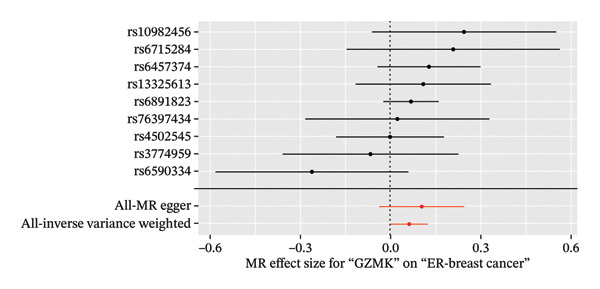
(f)

In the two‐sample MR analysis of TNBC, no heterogeneity was observed for SH2D2A (inverse variance weighting [IVW]: *p* = 0.4702), ADGRE5 (IVW: *p* = 0.4353), CD7 (IVW: *p* = 0.4688), or GZMK (IVW: *p* = 0.4325). The MR‐Egger method revealed that GZMK alone showed no horizontal pleiotropy in the context of the MR study of TNBC (GZMK: *p* = 0.5399; Figure 5(c) and Table S6). The MR‐Egger approach could not ascertain the horizontal pleiotropy of ADGRE5, SH2A2D, and CD7 because at least three SNPs were required for its application. Additionally, no heterogeneity was found among the nine SNPs regarding the causal effects between TNBC and GZMK (Figure 5(d)). Subsequently, we performed a leave‐one‐out analysis to determine whether the causal inference was influenced by any single factor (Figure 5(e)), which maintained a consistent positive association between genetically forecasted levels of GZMK and susceptibility to TNBC (Figure 5(f)). Therefore, based on current evidence, we believe that GZMK levels are more closely associated with a higher risk of TNBC. MR‐PRESSO analysis showed no evidence of horizontal pleiotropy or instrument distortion (global *p* > 0.05; no outliers; no distortion). These results support the validity of the IVs used for GZMK in our MR framework. We will enrich the instrument set with additional SNPs as they become available from larger GWAS to further improve power and reliability.

To bolster the credibility of our findings, we performed an analysis on a validation dataset (ieu‐a‐1135) employing MR to explore the relationship between four specific genes (*SH2D2A, ADGRE5, CD7*, and *GZMK*) and predisposition to TNBC. Our findings revealed a significant association between mutations in GZMK and increased TNBC risk, as evidenced by the MR‐Egger (OR = 1.3401, *p* = 0.0196), median weight (OR = 1.1476, *p* = 0.0137), and IVW methods (OR = 1.1504, *p* = 0.035) (Figure 6(a)). Additionally, mutations in SH2D2A were associated with increased susceptibility to TNBC (IVW: OR = 1.4227, *p* = 0.0137). However, MR analyses of ADGRE5 and CD7 showed no significant correlation with TNBC risk (*p* > 0.05). Similarly, inverse MR analyses revealed no significant correlation when TNBC was considered the exposure and the *GZMK* gene was considered the outcome (Figure 6(b)). The comprehensive analysis included scatter and funnel plots, as shown in Figures 6(c) and 6(d). The MR‐Egger *p*‐value used for testing heterogeneity was 0.7223, indicating the absence of heterogeneity. Furthermore, the MR‐Egger test for pleiotropy resulted in a *p*‐value of 0.0563245 (Table S7), indicating that no direct impact exists on outcome severity. Leave‐one‐out analysis, coupled with the forest plot, consistently corroborated a robust positive correlation between genetically deduced increases in GZMK expression and an increased risk of TNBC within the validation set (Figures 6(e) and 6(f)).

FIGURE 6Bidirectional MR estimates for the casual associations between key genes and TNBC risk. (a) Forest plot depicting the association of key genes with TNBC risk based on the EBI database (ieu‐a‐1135); an odds ratio greater than 1.0 indicates a factor was associated with a higher risk of TNBC, and odds ratio lower than 1.0 indicates a correlation with a lower risk of TNBC. (b) Bidirectional Mendelian randomization estimates for the casual associations between key genes and TNBC risk. (c) Funnel plots to visualize the overall heterogeneity of MR estimates for the effect of GZMK on TNBC risk. (d and e) MR scatter plot and a leave‐one‐out plot showing the effect of GZMK on TNBC. (f) Forest plot of MR analysis for nine SNPs of GZMK. EBI: European Bioinformatics Institute; MR: Mendelian randomization; SNP: single‐nucleotide polymorphism; TNBC: triple‐negative breast cancer.

**Figure:**
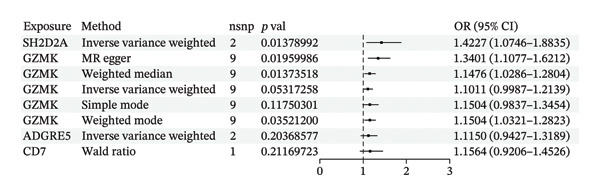
(a)

**Figure:**
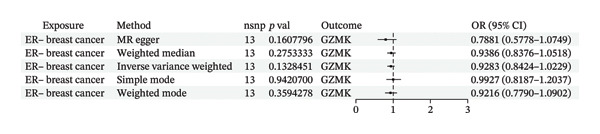
(b)

**Figure:**
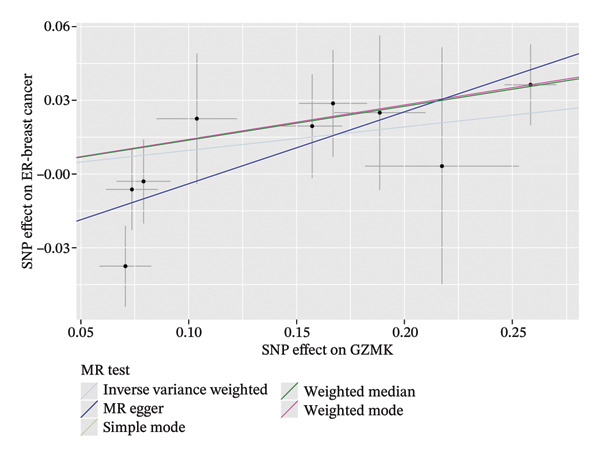
(c)

**Figure:**
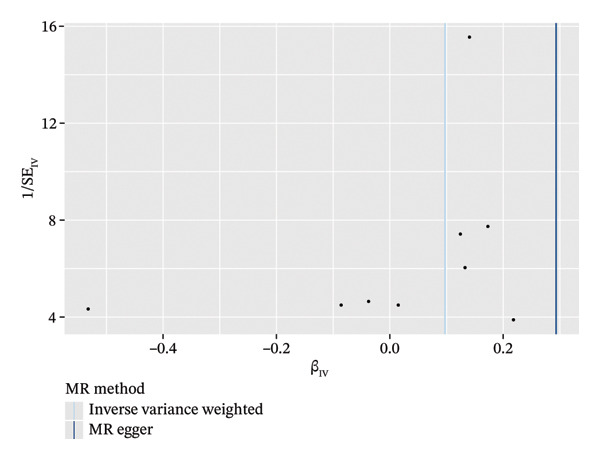
(d)

**Figure:**
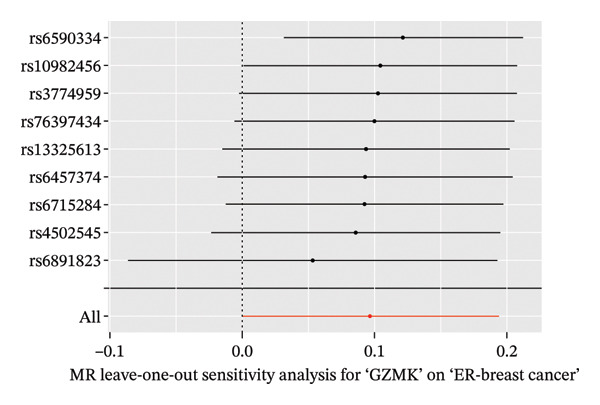
(e)

**Figure:**
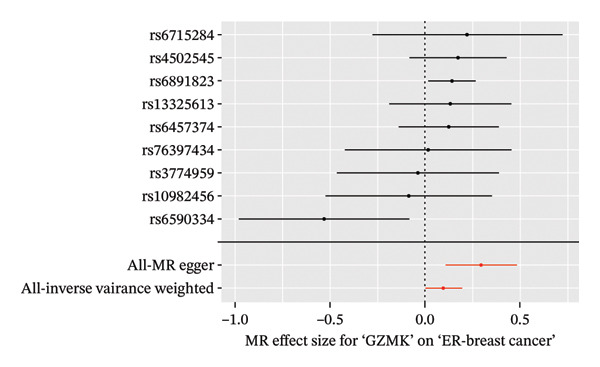
(f)

### 3.6. Colocalization Analysis for GZMK Gene and TNBC

Through MR analysis, we revealed a causal link between GZMK and TNBC. Subsequently, we conducted a colocalization analysis focusing on TNBC within the genomic vicinity of the drug target genes, encompassing a span of ±1 Mb pairs around GZMK. The results of this in‐depth examination are presented in Table S8. Our findings offer compelling evidence for colocalization between TNBC and the genomic territory of the *GZMK* gene, with an overwhelmingly high posterior probability, indicating a shared causal variant (TNBC: 0.9999 in GZMK). This statistical marker underscores the profound genetic intersection that may illuminate the pathways through which GZMK influences the onset and progression of TNBC.

We visually represented these findings using regional association plots that illustrate the interaction between the variants within GZMK and TNBC (Figure 7). By comparing the strengths of these associations, we identified several SNPs that exhibited substantial associations between the two phenotypes. Particular SNPs intricately linked with eQTLs within the *GZMK* gene—for instance, rs6891823—revealed a notable correlation in the GWAS focused on assessing the risk of TNBC. This graphical depiction not only enhances our understanding of the genetic landscape but also reinforces the potential of GZMK as a pivotal point of convergence in the genetic etiology of TNBC, thereby opening avenues for targeted therapeutic interventions. Additionally, we conducted a Steiger test to determine the direction of causation. These results corroborate the original notion that alterations in GZMK expression might affect susceptibility to TNBC (Table S9). Our study indicates a possible cause‐and‐effect association between GZMK and the risk of TNBC.

FIGURE 7Regional association map of the GZMK genetic region on chromosome 5, centered around the lead SNP rs6891823. SNP: single‐nucleotide polymorphism.

**Figure:**
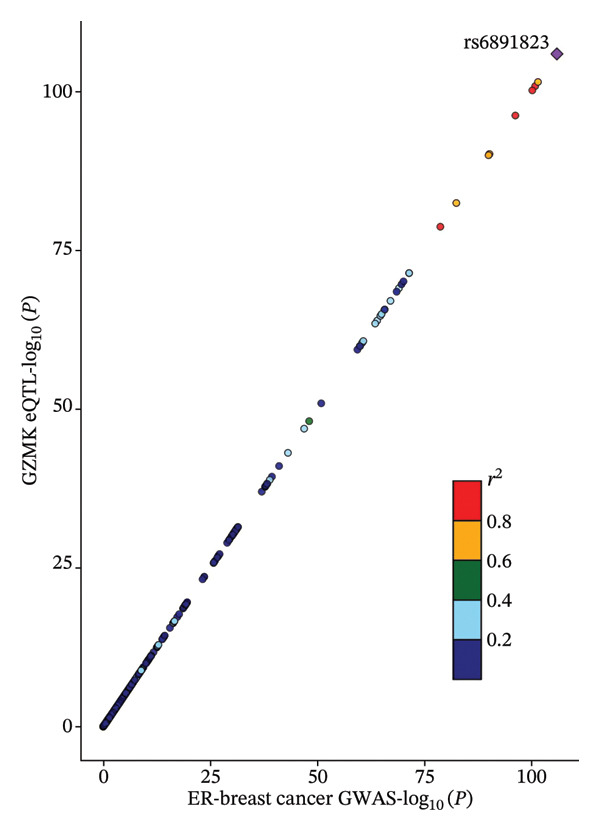
(a)

**Figure:**
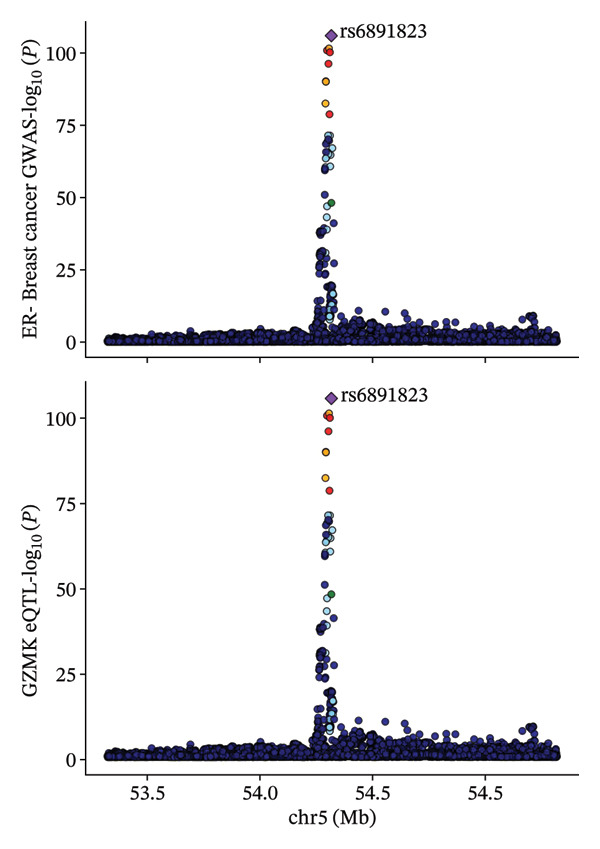
(b)

### 3.7. GZMK Has Been Identified as a Crucial Factor in T‐Cell Efficiency in TNBC

The UMAP algorithm was used to downscale and visualize single‐cell transcriptome data, identifying different cell subpopulations. Figure 8(a) shows the expression patterns of four significant marker genes across various T‐cell types. Subsequently, the feature plot tool was used to highlight the expression of T‐cell GZMK, ADGRE5, SH2D2A, and CD7, providing possible leads for further functional analysis (Figure 8(b)). Intercellular communication patterns differed between the GZMK+ CD8_CM and GZMK‐ CD8_CM cell subsets, with increased cellular signaling activity and a more complex array of ligand–receptor contacts observed in the GZMK+ CD8_CM fraction, indicating a higher degree of communication complexity in this group (Figure 8(c)). Moreover, comparing the enhanced signaling pathways of GZMK+ CD8_CM and GZMK‐ CD8_CM cells revealed the involvement of the CCL3‐CCR1 and CCL3L1‐CCR1 signaling pathways in cellular communication in TNBC (Figure 8(d)). This suggests that GZMK+ CD8_CM cells play a vital role in both pathways. Additionally, using CellPhoneDB, we discovered frequent interactions between GZMK + CD8_CM and other cell types (Figure 8(e)), with macrophages as the primary interactors, followed by monocytes and epithelial cells (Figures 8(f) and 8(g)). We specifically examined the cell‐to‐cell interactions between GZMK+ CD8_CM and endothelial cells. The most frequent interaction between GZMK+ CD8_CM and epithelial cell types involved the MIF‐(CD74+ CD44) signaling pathway (Figure 8(h)), consistent with our previous findings.

FIGURE 8Single‐cell transcriptomic dissection exposes GZMK as a crucial determinant of T‐cell efficacy in TNBC. (a) Expression pattern of key genes across different cell subpopulations. (b) UMAP dimensionality reduction visualizing cell subgroups with differential expressions of specific key genes. (c) Intercellular communication analysis between GZMK + CD8_CM and GZMK‐CD8_CM cell subgroups using the CellChat tool. (d) Differential signaling pathways enriched in communications involving GZMK + CD8_CM cells compared to others. (e and f) Intercellular communication analysis between GZMK + CD8_CM and GZMK‐CD8_CM cell subgroups using the CellPhoneDB tool. (g) Heatmap showing the number of potential ligand–receptor pairs among the predicted cell types. (h) Ligand–receptor pairs detected with CellPhoneDB are shown in a bubble plot. TNBC: triple‐negative breast cancer; UMAP: Uniform Manifold Approximation and Projection.

**Figure:**
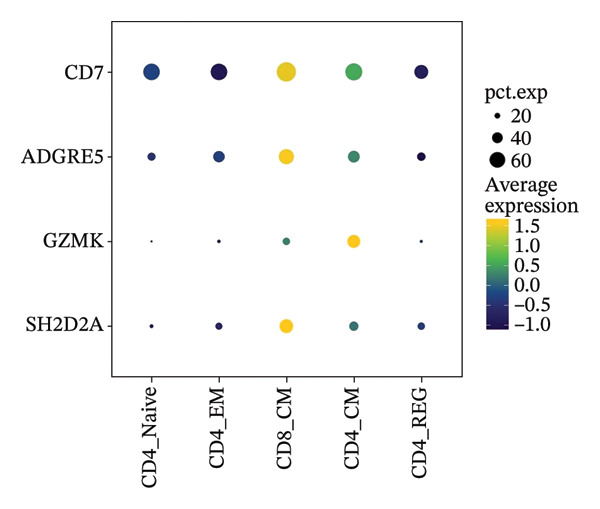
(a)

**Figure:**
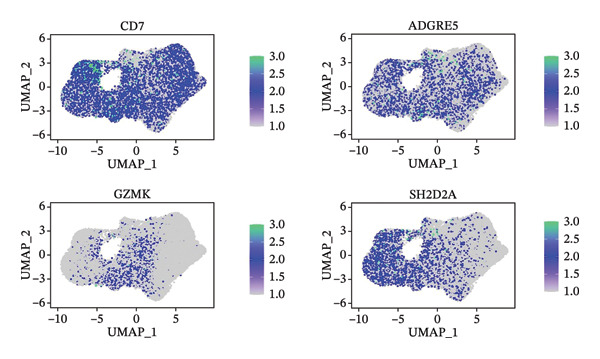
(b)

**Figure:**
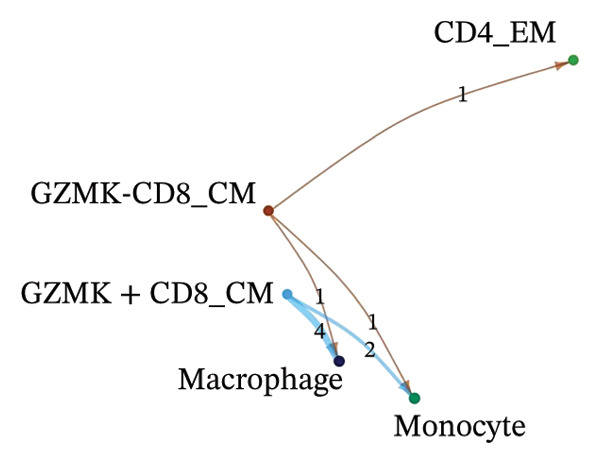
(c)

**Figure:**
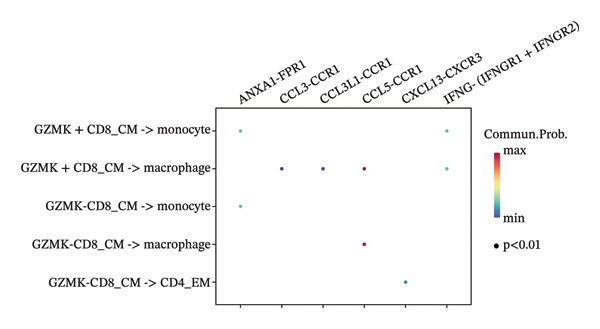
(d)

**Figure:**
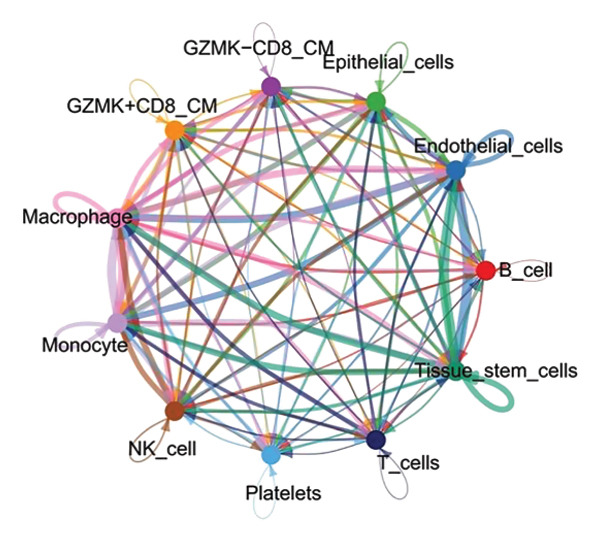
(e)

**Figure:**
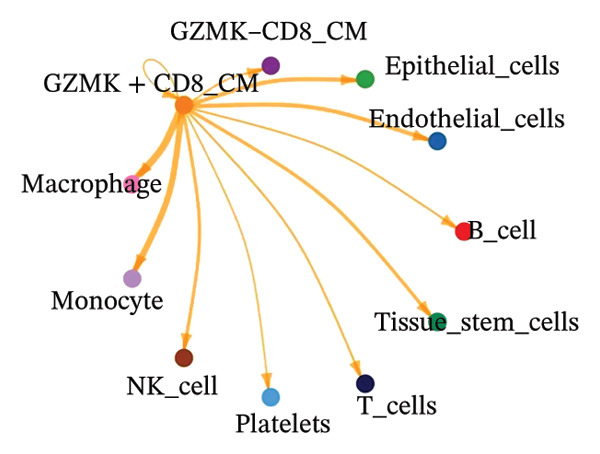
(f)

**Figure:**
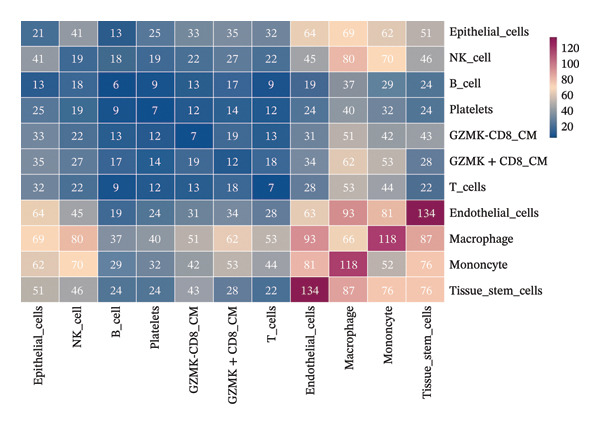
(g)

**Figure:**
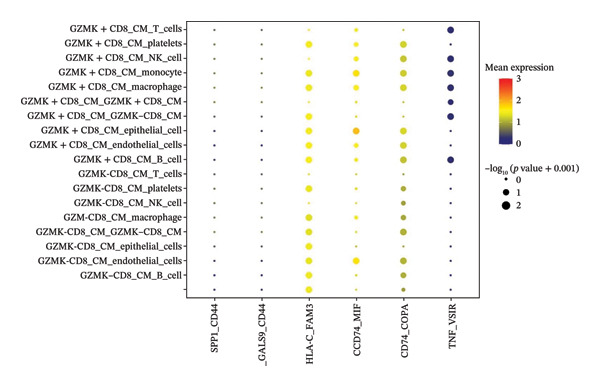
(h)

### 3.8. Single‐Cell Transcriptomic Insights Unveil the Critical Function of GZMK in T‐Cell Metabolism Within TNBC

The analysis of metabolic pathways identified specific activation stages of T cells, and the intricacies of intracellular metabolic activity were investigated using the scMetabolism tool. Analysis of metabolic pathways showed that patients with TNBC had lower levels of T‐cell metabolism (Figure 9(a)), particularly in CD8_CM cells, than in normal and senescent samples (Figure 9(b)). Significant differences were observed in key metabolic pathways. The GZMK+ CD8_CM group exhibited thiamine metabolism, nicotine and nicotinamide metabolism, and glycosphingolipid biosynthesis (lacto and neolacto series) (Figure 9(c)), which sustain malignant cells and modulate the TME, influencing disease progression and therapeutic responses [49–51], and the corresponding results were also verified in the bulk data (Figure S2). Understanding these pathways in detail paves the way for novel interventions capable of disrupting the metabolic dependencies of TNBC cells, thereby offering new avenues for treatment strategies. Subsequently, slingshot cell trajectory analysis indicated dynamic changes in T cells as the disease progressed (Figure 9(d)). The temporal regulation of gene activity was intricately mapped using pseudotime analysis, delineating the transcriptional shifts accompanying cellular maturation or dynamic expression patterns (Figure 9(e)).

FIGURE 9Single‐cell transcriptomic insights illuminate the pivotal role of GZMK in regulating T‐cell metabolism within TNBC. (a) Profiling of metabolic pathway engagement across diverse T‐cell populations within old and TNBC scRNA‐seq datasets. (b) Comparative analysis of metabolic pathway activities of CD8_CM in control (CT) and TNBC groups. (c) Metabolic pathway analysis showing the activation states of T cells, revealing differences in metabolic activities between GZMK + CD8_CM and GZMK‐CD8_CM cell subgroups. (d) Cell trajectory analysis from primary to mature T‐cell states using the Slingshot tool. (e) Temporal gene activity (on/off status) maps capturing gene expression fluctuations throughout cellular development or transcriptional reprogramming phases. (f) Scatter diagram illustrating the correlation between “GZMK” gene expression and cellular developmental timelines (pseudotime). (g) Enrichment analysis of DEGs between GZMK + CD8_CM and GZMK‐CD8_CM cell subgroups. (h) Heatmap showing the differences in expression levels of key genes in TNBC and normal tissues based on bulk‐seq datasets (GSE38959, GSE45827, and GSE65194). (i) Box plots showing the differential expression of GZMK in TNBC and normal tissues in bulk‐seq datasets (GSE38959, GSE45827, and GSE65194). DEGs: differentially expressed genes; TNBC: triple‐negative breast cancer.

**Figure:**
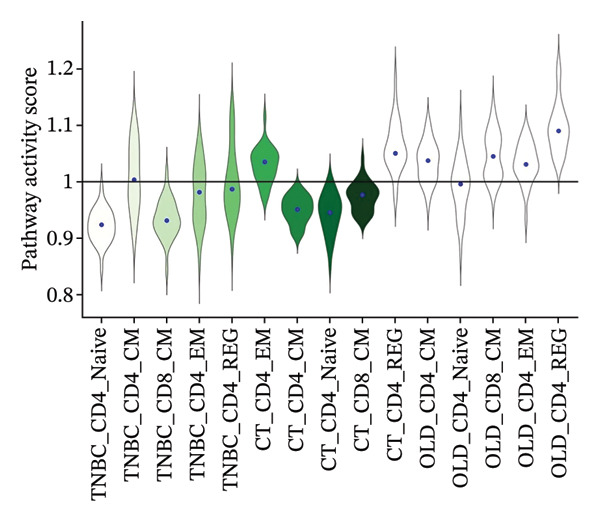
(a)

**Figure:**
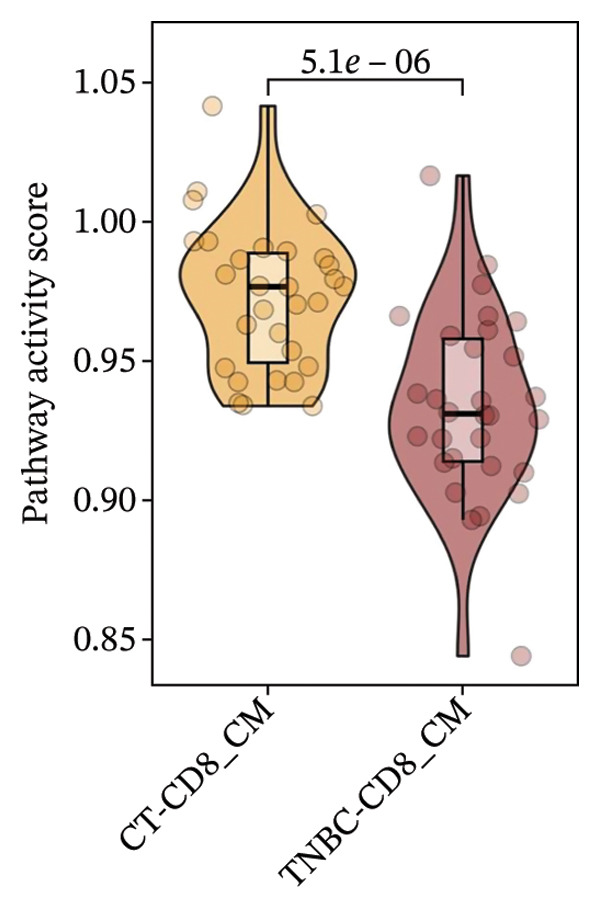
(b)

**Figure:**
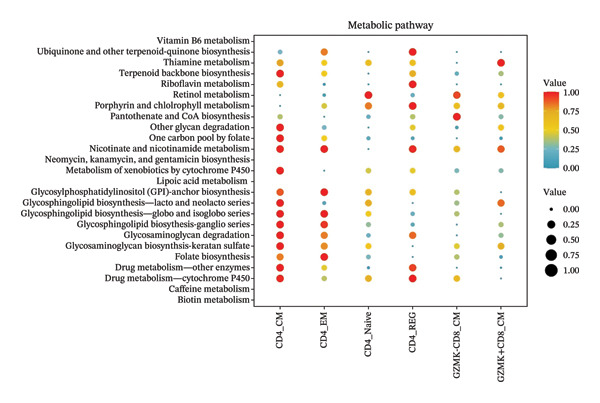
(c)

**Figure:**
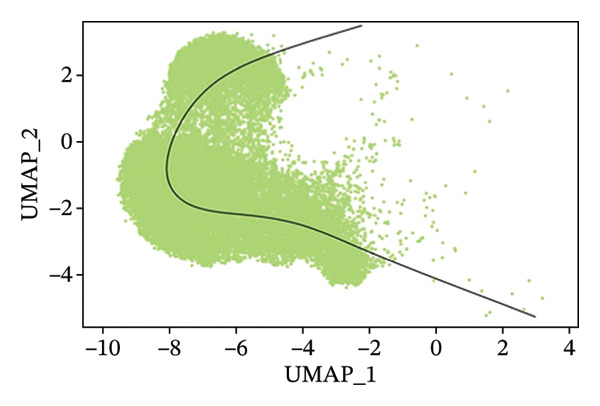
(d)

**Figure:**
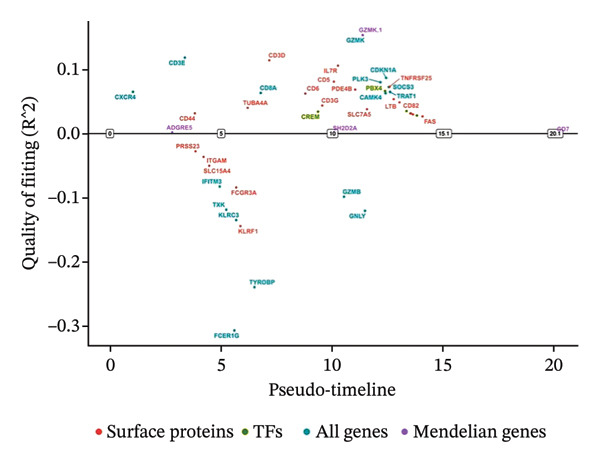
(e)

**Figure:**
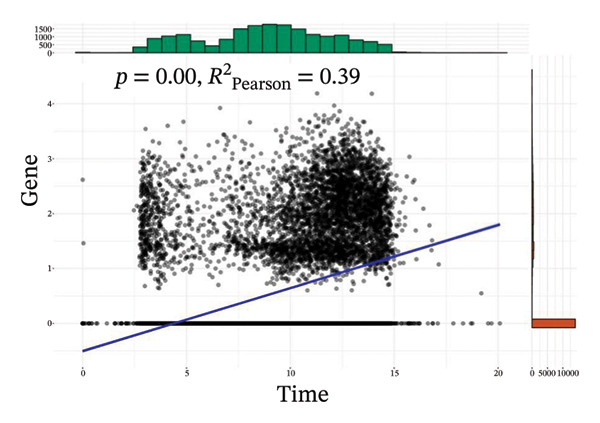
(f)

**Figure:**
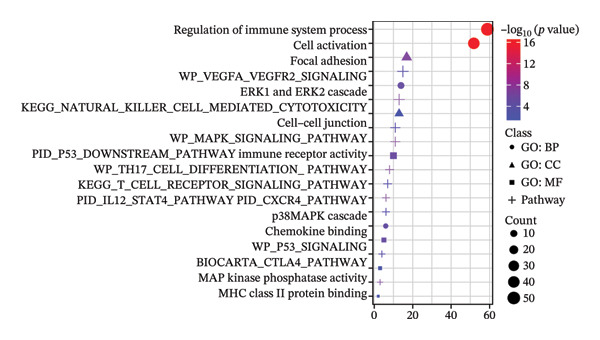
(g)

**Figure:**
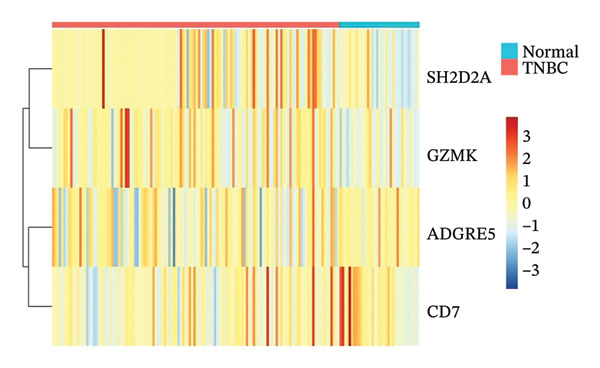
(h)

**Figure:**
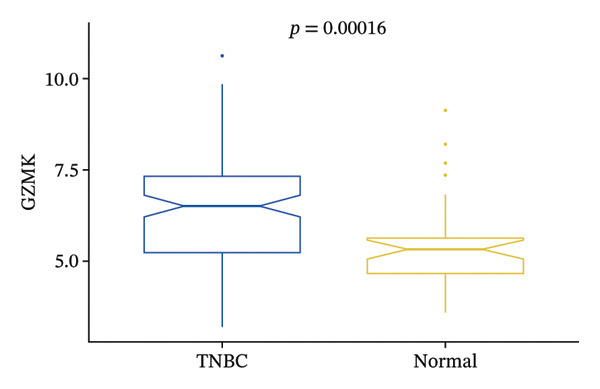
(i)

Scatter plot analysis showed a significant positive correlation between GZMK expression and pseudotime progression (Pearson *r* = 0.39, *p* < 0.001), suggesting an increase in GZMK expression over the pseudotime course (Figure 9(f)). Moreover, comparative gene expression profiling of GZMK‐positive CD8_CM and GZMK‐negative CD8_CM T‐cell subsets revealed alterations in the expression patterns of several genes implicated in susceptibility. Enrichment analysis of these variably expressed genes indicated an enhanced regulatory role of GZMK+ CD8_CM cells in several biological processes, particularly GO:0002252 related to the immune effector process and GO:0050778 related to broader immune response. Additionally, this analysis encompassed pathways such as GO:0071345, which involves cellular response to cytokine stimuli, and GO:0002366, highlighting leukocyte activation within the immune response spectrum (Figure 9(g)).

Integrating transcriptomic datasets from TNBC with control samples from healthy tissues across three GEO databases (GSE38959, GSE45827, and GSE65194) showed significant differential expression of GZMK when comparing patients with TNBC to the nonaffected cohort (Figures 9(h) and 9(i)). This observation corroborated the validity and empirical robustness of our single‐cell transcriptomic assessment.

### 3.9. *In Vitro* Experiments Validated That GZMK has Tumor‐Promoting Effects

**FIGURE 11:**
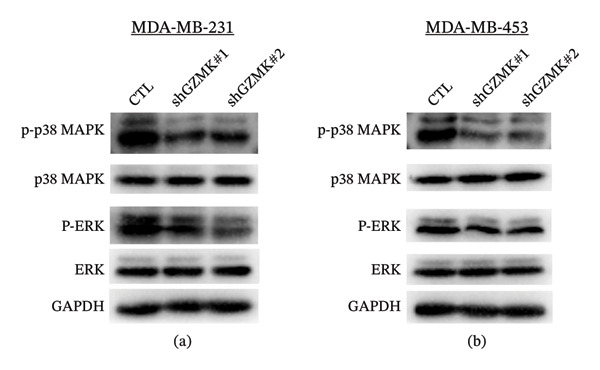
Knockdown of GZMK inhibits the MAPK/ERK signaling pathway in TNBC cell lines. (a) MDA‐MB‐453 and (b) MDA‐MB‐231 cell. ERK, extracellular signal‐regulated kinase; p38 MAPK, p38 mitogen‐activated protein kinase (MAPK); p‐, phosphorylated. GAPDH acted as an internal control.

Subsequently, a series of *in vitro* experiments were conducted to explore the potential role of GZMK in the development and progression of TNBC. Using lentiviral vectors to mediate shGZMK infection in the TNBC cell lines MDA‐MB‐453 and MDA‐MB‐231, we successfully achieved significant suppression of GZMK expression at the protein level (Figures 10(a) and 10(b)). MTT and colony formation assays demonstrated that GZMK downregulation significantly reduced the proliferation rate and colony‐forming ability of MDA‐MB‐453 and MDA‐MB‐231 cells (Figures 10(c) and 10(d)). Furthermore, 5‐ethynyl‐2′‐deoxyuridine (EdU) assays confirmed that GZMK downregulation markedly slowed cell growth (Figures 10(e) and 10(f)). To investigate the role of GZMK in cell invasion and metastasis, we assessed changes in the invasive and migratory capacities of stable GZMK‐knockdown cell lines. Wound healing assays revealed that GZMK knockout significantly impaired the migration of MDA‐MB‐453 and MDA‐MB‐231 cells (Figures 10(g) and 10(h)). This finding was further supported by Transwell assays, which showed a marked decrease in their invasion and migration abilities (Figures 10(i) and 10(j)). These results strongly suggest that GZMK plays a critical regulatory role in promoting TNBC cell proliferation, invasion, and migration, potentially acting as an oncogene in the progression of TNBC (see Figure 11).

FIGURE 10Knockdown of GZMK inhibited the proliferation and migration of TNBC cells *in vitro*. (a and b) Western blotting of GZMK protein levels in MDA‐MB‐453 and MDA‐MB‐231 cells infected with shRNAs. (c) MDA‐MB‐453 and MDA‐MB‐231 cell proliferation after knockdown of GZMK by MTT assay. (d–f) Representative results of the colony formation and EdU assays (scale bar: 100 μm) in MDA‐MB‐453 and MDA‐MB‐231 cells after GZMK‐sh1 or GZMK‐sh2 transfection. (g–j) Representative images of TNBC cell migration ability as shown by wound healing assays (g and h) and Transwell migration assay (I,J). ^∗∗^ *p* < 0.01; ^∗∗∗^ *p* < 0.001. TNBC: triple‐negative breast cancer.

**Figure:**
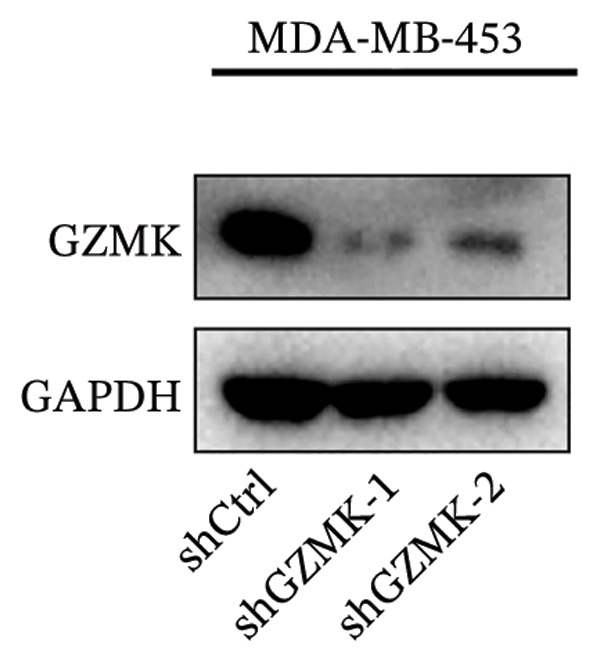
(a)

**Figure:**
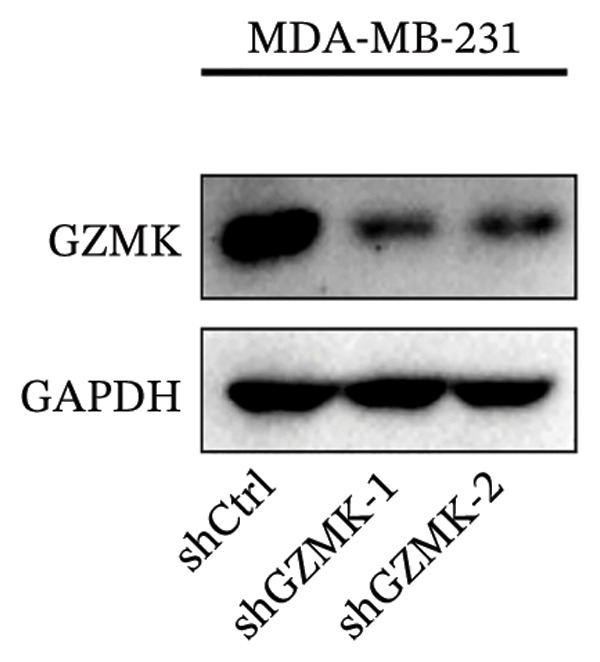
(b)

**Figure:**
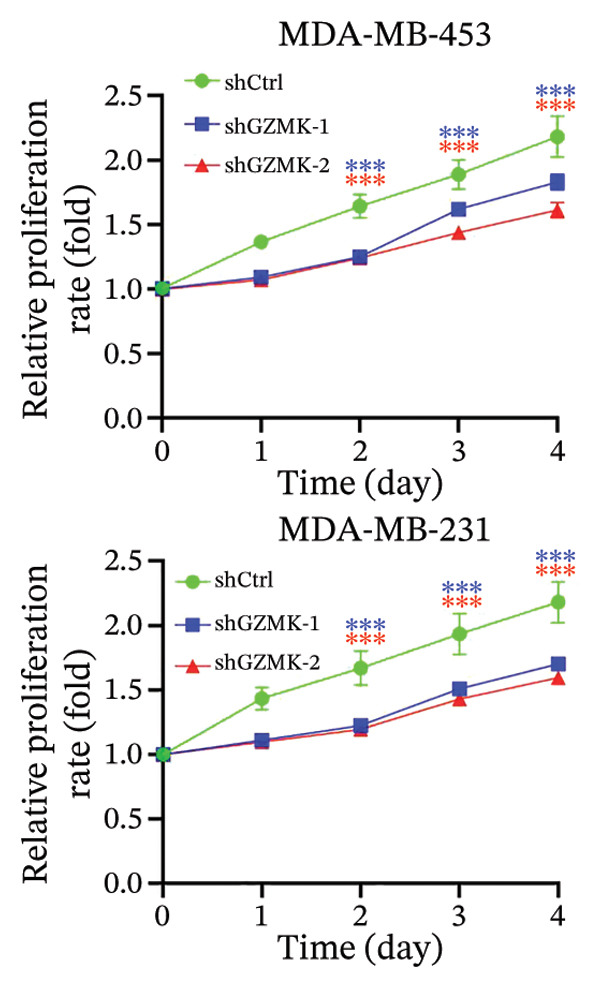
(c)

**Figure:**
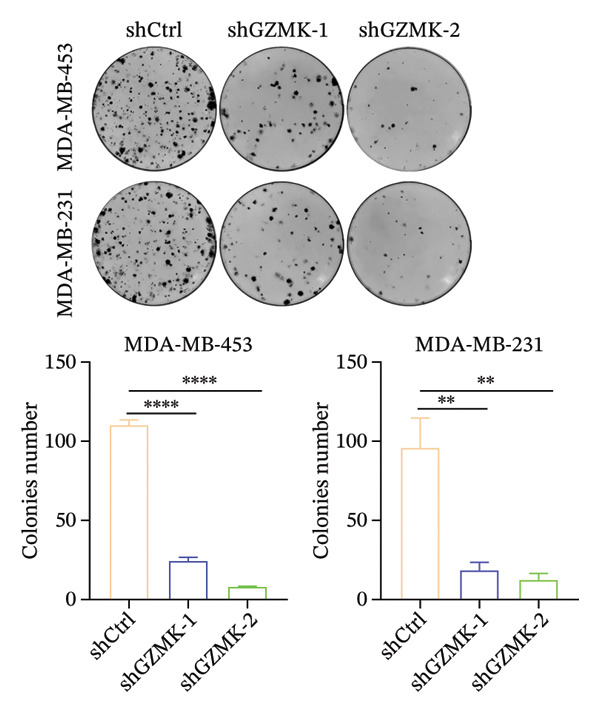
(d)

**Figure:**
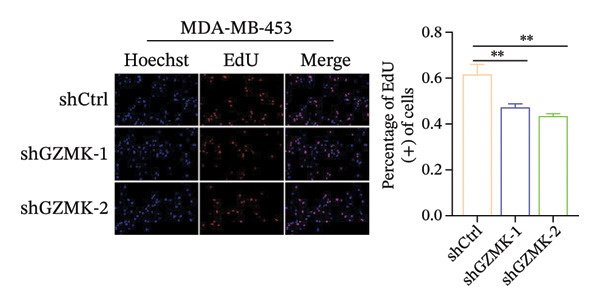
(e)

**Figure:**
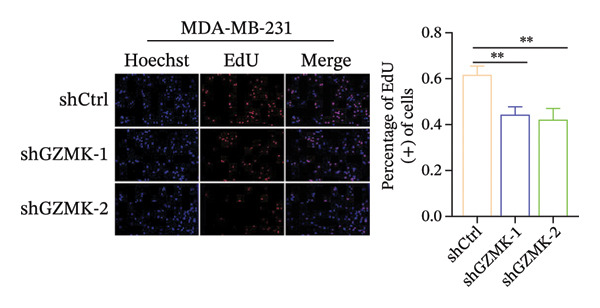
(f)

**Figure:**
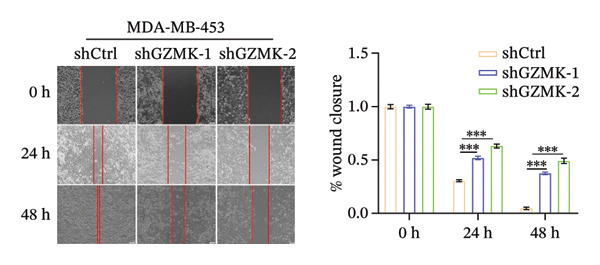
(g)

**Figure:**
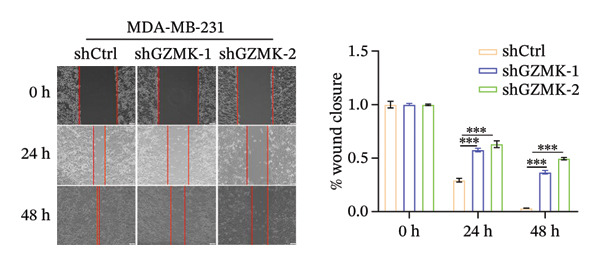
(h)

**Figure:**
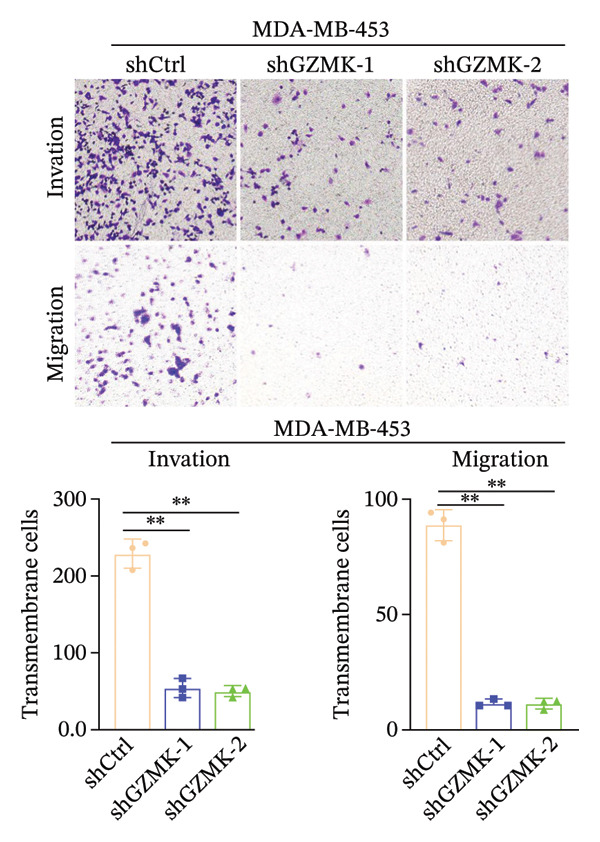
(i)

**Figure:**
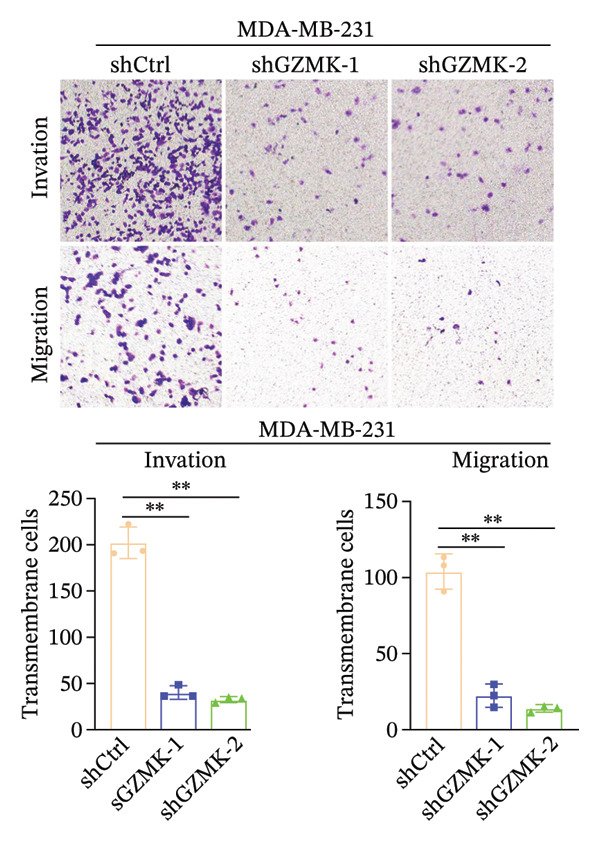
(j)

In MDA‐MB‐231 and MDA‐MB‐453 TNBC cell lines, GZMK knockdown did not alter total ERK1/2 or total MAPK protein levels. However, p‐ERK1/2 levels were significantly reduced upon GZMK knockdown, indicating that GZMK promotes MAPK signaling by enhancing ERK1/2 phosphorylation rather than by increasing total kinase abundance (Figure S3).

## 4. Discussion

In this comprehensive analysis, we explored the landscape of immune cell groups among older individuals, people with TNBC, and those in good health using scRNA‐seq. Our research supports the existence of various immune cell types, including T cells, epithelial cells, endothelial cells, macrophages, and B cells, consistent with previous studies [52, 53]. Notably, CD8+ T cells have emerged as pivotal agents of antitumor immunotherapy, and their abundance correlates with significantly enhanced survival rates in patients with TNBC due to their direct engagement with and annihilation of cancer cells through MHC I peptide presentation [54–57]. The infiltration of T cells into tumors is a crucial indicator for predicting the efficacy of programmed cell death protein 1 blockade therapies and is closely linked to favorable prognostic outcomes. Moreover, the interaction between tumor PD‐L1 and the suppression of antitumor activities of CD8+ T cells underscores the complexity of immunological responses in cancer [58].

Our investigation found that all TNBC groups had fewer T cells than healthy controls. The lack of tumor‐infiltrating lymphocytes in TNBC is believed to be the principal factor contributing to the diminished effectiveness of immunotherapy for this cancer variant, as it hinders the stimulation of antitumor immunity [59]. This finding is consistent with previous studies indicating that the immunosuppressive TNBC microenvironment is due to the nonimmunogenic nature of tumor cells [60, 61] and their inability to activate anticancer immunity [62].

Despite the acknowledged significance of CD8+ T cells in combating tumors, our study highlights the pressing need for a deeper exploration of their specific functional mechanisms in the context of TNBC. A noteworthy observation from our investigation was the diminished presence of CD8_CM in patients with TNBC, potentially indicative of compromised immune capabilities. The metabolic exhaustion experienced by tumor‐infiltrating CD8+ T cells, culminating in cellular dysfunction, is a critical aspect of this phenomenon [63–65]. Therefore, the altered dynamics of CD8_CM cells may reflect their pivotal role in orchestrating immune responses against diseases.

Moreover, our investigation involved functional enrichment analysis, which elucidated the distinctive pathways inherent to various cellular subpopulations, providing insight into their physiological roles. Using single‐cell trajectory analysis, we predicted the pathways of T cells from their initial stages of development until they reached full maturation. This approach provides novel insights into the mechanisms underlying T‐cell growth and activation. Significantly, the analysis of intercellular communication networks revealed heightened interactions, especially between CD8_CM cells and macrophages, monocytes, and endothelial cells in patients with TNBC, with a pronounced emphasis on the MIF–CD74 pathway. This observation suggests a crucial role for the MIF pathway in disease progression.

The prognostic value of CD8+ T cells in solid tumors is well‐documented [66–69], with elevated levels of memory CD8+ T cells being associated with the inhibition of early metastatic spread and improved survival outcomes [70, 71]. However, the tumor immune landscape exhibits considerable heterogeneity. Research by Tiberti et al. demonstrated a significant interaction between GZMK‐expressing CD8+ EM cells and neutrophils in nonmetastatic colorectal cancer, which contributed to tumor advancement. This interplay is a hallmark of colorectal cancer that directly influences patient outcomes [72].

Our findings revealed that throughout CD8_CM cell development, the expression of GZMK increased, establishing a causal link with TNBC progression. GZMK, a trypsin‐like protein belonging to the granzyme family, is produced by CTLs and NKTs [73–76]. Although the role of GZMK is not fully understood, research conducted in laboratory settings has shown that GZMK, in conjunction with perforin, produces ROS and disrupts mitochondrial activity to cause nonapoptotic cell death [77]. Additionally, GZMK may cleave the oncogene p53, which sensitizes tumor cells to apoptosis [78] and induces the activation of the ERK1/2 and p38 MAPK signaling pathways, resulting in a notable increase in fibroblast growth in individuals with sepsis and acute lung inflammation [79]. ERK1/2 [80, 81] and p38 MAPK [82–84] are widely recognized oncogenic pathways crucial for the development and progression of TNBC. These results indicate that GZMK might function via the ERK1/2 or P38 MAPK pathways and could be a valuable target for TNBC treatment in the upcoming years. GZMK promotes TNBC progression by increasing ERK1/2 phosphorylation and activating MAPK signaling, with downstream effects on tumor behavior. Although our *in vitro* knockdown in established cell lines is compelling, it may not capture TNBC heterogeneity; orthogonal validation in clinically relevant models is needed. We will perform CRISPR/Cas9 knockouts and rescues in additional TNBC models and directly assess ERK1/2 and p38 MAPK activity to validate the GZMK–MAPK axis. Consistent with transcriptomic and single‐cell data, GZMK appears to drive MAPK programs that promote progression. To establish causality, we will implement MEK/ERK inhibitor and constitutively active ERK rescue experiments, conduct time‐resolved phosphoproteomics to map activation kinetics, and validate the axis *in vivo*. We will also explore upstream receptors or mediators, including paracrine signals from the TME. Supporting data and detailed methods will accompany these analyses.

The findings of this study align with emerging literature indicating that CD8+ T‐cell gene signatures and their functional states have predictive value for prognosis and immunotherapy responses across cancers [85]. Although these studies do not directly address GZMK+ CD8_CM in TNBC, GZMK—an important effector molecule in CD8+ T cells—may comprise part of such predictive signatures. Conversely, signals from viral interactions can shape tumor stemness and T‐cell infiltration, underscoring how extrinsic cues modulate immune cell subsets; endothelial and MIF‐related signaling axes in the TNBC microenvironment may also influence immune infiltration [86, 87]. Furthermore, a CD8+ T cell–derived signature has shown prognostic value in clear cell RCC, supporting cross‐cancer relevance of CD8+ T‐cell states as biomarkers [88]. Taken together, the potential role of GZMK + CD8_CM in TNBC likely reflects a composite signaling module linked to immunotherapy sensitivity, vascular–immune crosstalk, and microenvironment remodeling, which warrants validation in TNBC‐specific cohorts with multimodal evidence to establish clinical translational potential.

We provide cautious mechanistic hypotheses to guide future experiments, without overasserting causality. Potential signaling axes for GZMK‐expressing CD8+ T cells include the ERK1/2 and p38 MAPK pathways. Mechanistic work should include *in vitro* co‐culture systems to assess how GZMK‐expressing CD8+ T cells influence TNBC cell signaling and invasion, *in vivo* models to evaluate how GZMK modulation affects tumor growth and TME composition, and multiparameter profiling (proteomics, phosphoproteomics) to map downstream signaling alterations in TNBC cells exposed to GZMK‐rich T‐cell environments. Overall, while our multiomics approach provides converging evidence for a role of GZMK + CD8+ T cells in TNBC, conclusions should be interpreted in light of the above limitations. We view this work as a stepping stone toward deeper mechanistic dissection and clinical translation, and anticipate that ongoing and future studies will validate and extend these findings across broader patient populations and experimental models. Our findings highlight GZMK as a potential immunobiomarker and therapeutic target within the TNBC immune microenvironment. To translate these observations toward clinical practice, future work could include developing GZMK‐based assays to stratify patients for immunotherapy regimens or combination strategies; conducting preclinical modeling to test whether modulating GZMK‐expressing CD8+ T cells alters TNBC growth, metastasis, or response to PD‐1/PD‐L1 blockade; designing early‐phase clinical trials that combine GZMK pathway modulation with established immunotherapies, with endpoints including TME remodeling, T‐cell activity, and objective response rates; and integrating multiomics data to identify patient subgroups most likely to benefit from GZMK‐targeted approaches, considering factors such as age and tumor stage where data permit.

Despite the insights offered, our study had some limitations, notably its modest sample size, underscoring the need for validation through broader studies. Future research should aim to empirically ascertain the roles of pivotal genes in disease genesis and progression, supplemented by animal model studies to elucidate their molecular and regulatory mechanisms. Although integrating multiple scRNA‐seq and bulk RNA‐seq datasets strengthens cross‐validation, several limitations warrant emphasis. Age distribution and TNBC staging information are not uniformly balanced across datasets, which may confound disentangling aging‐related effects from tumor‐stage effects on the immune milieu. Technical heterogeneity arises from disparate scRNA‐seq platforms, preprocessing pipelines, and quality‐control criteria; while Harmony was used to mitigate batch effects, residual technical variation may still influence clustering, trajectory inference, and cell–cell communication analyses. Subpopulation definitions depend on reference annotations and computational methods (e.g., SingleR, UMAP), and misannotation or overclustering could bias conclusions about GZMK+ CD8+ T cells. Public datasets often lack uniform clinical metadata (e.g., treatment history, exact TNBC subtypes), limiting the ability to disentangle treatment‐ or subtype‐specific effects on GZMK expression and TME interactions. Finally, external validity may be limited: Conclusions drawn from European‐ancestry GWAS and published scRNA‐seq cohorts may not fully generalize to diverse populations; future work should assess generalizability across ancestries and independent cohorts.

MR provides a framework to infer causality, but relies on three core assumptions (strong association of SNPs with exposure, independence from confounders, and no horizontal pleiotropy). While we applied multiple sensitivity analyses, more nuanced limitations deserve emphasis: First, subtype heterogeneity is a concern, as our exposure GWAS (ieu‐a‐1128 and ieu‐a‐1135) largely reflect ER− breast cancer contexts and may not perfectly capture TNBC‐specific genetics, with differences between TNBC and broader ER− disease potentially influencing causal estimates; second, pleiotropy and pathways remain possible, as some instruments might affect TNBC risk through mechanisms other than GZMK expression or the studied senescence‐related biology, biasing estimates; third, instrument strength and number may be limiting, given that for several genes only a subset of SNPs meet genome‐wide significance, which can limit statistical power and increase susceptibility to weak‐instrument bias; fourth, although we assessed horizontal pleiotropy and outliers using MR‐PRESSO (with results alongside the original MR analyses provided in Supporting Information) and, where possible, augmented the SNP set to improve robustness, residual bias cannot be excluded; finally, other sources of bias, including sample overlap, population structure, and GWAS data limitations, may affect generalizability beyond the studied ancestry.

In conclusion, our study elucidates the complex interplay among immune cell composition, GZMK gene expression, aging, and TNBC, proposing several hypotheses for further exploration. This study enhances our understanding of TNBC’s molecular basis and highlights novel molecular targets for therapeutic intervention, thereby significantly contributing to precision medicine research in TNBC.

## Author Contributions

Conceptualization: Yidan Xu, Dan Zhou, Zhong Ouyang, Liangxi Xie, Beibei Xu, Yongcheng Su, and Tianhui Hu; data curation: Dan Zhou, Yidan Xu, Yongcheng Su, Beibei Xu, and Zhong Ouyang; formal analysis: Dan Zhou, Yongcheng Su, Beibei Xu, and Tianhui Hu; investigation, Yidan Xu, Dan Zhou, and Beibei Xu; methodology: Yidan Xu and Wenqing Zhang; resources: Yidan Xu and Yongcheng Su; software: Jiachen Zhu, Beibei Xu, and Miaomiao Ma; validation: Miaomiao Ma, Jiachen Zhu, and Wenqing Zhang; visualization: Beibei Xu and Yongcheng Su; writing–original draft: Yidan Xu, Yongcheng Su, Beibei Xu, Liangxi Xie, Zhong Ouyang, and Tianhui Hu; and review and editing: Yidan Xu, Liangxi Xie, Wenqing Zhang, and Tianhui Hu.

## Funding

This work was supported by grants from the Natural Science Foundation of Xiamen City of China (No. 3502Z202573104), the Natural Science Foundation of Fujian Province of China (No. 2023J011660), the Scientific Research Startup Fund of Xiamen City University (Nos. 20105026 and 20105027), the Shenzhen Science and Technology Program (JCY20220530143406015), the National Natural Science Foundation of China (82204424), the Xiamen Science and Technology Project (3502Z20227306, 3502Z20227157), and the Scientific Research Foundation of Xiang And Biomedicine Laboratory (2023XAKJ0101023).

## Disclosure

All authors have read and agreed to the published version of the manuscript.

## Conflicts of Interest

The authors declare no conflicts of interest.

## Supporting Information

Additional supporting information can be found online in the Supporting Information section.

## Supporting information


**Supporting Information 1** Supporting Information 1: Figure S1. Quality control (QC) of the scRNA‐seq data. Figure S2. The correlation between metabolic pathway and GZMK expression level by gene set enrichment analysis (GSEA) based on TCGA–TNBC cohort.

**Supporting Information 2** Supporting Information 2: Table S1. Enrichment analysis of DEGs in the epithelial cell subpopulation (Top 10). Table S2. Enrichment analysis of DEGs in the five T‐cell subpopulations (Top 10). Table S3. GO enrichment analysis on each subcluster. Table S4. Marker genes of different T‐cell types. Table S5. 40 key genes that distinguish CD8_CM from other cell types and T‐cell subtypes. Table S6. Mendelian randomization estimates for key genes on TNBC based on the EBI database (ieu‐a‐1128). Table S7. Mendelian randomization estimates for key genes on TNBC based on the EBI database (ieu‐a‐1135). Table S8: Colocalization probabilities between GZMK and TNBC. Table S9. Result of the Steiger test in two‐sample MR estimates of effects of key genes on TNBC.

**Supporting Information 3** Supporting Information 3: Supporting Data 1. Marker genes of different cell types. Supporting Data 2. Marker genes of different cell types after downsampling. Figure R1. Single‐cell RNA sequencing analysis of breast cancer tissue before and after downsampling. (A) Elbow plots were used to determine the number of principal components for subsequent analyses. (B) The UMAP algorithm was applied to the first 15 principal components for dimensionality reduction to obtain 25 cell clusters. (C) Annotation of the 25 clusters using the SingleR R package, categorizing them into 10 cell types. (D) Elbow plots were used to determine the number of principal components for subsequent analyses after downsampling. (E) The UMAP algorithm was applied to the first 15 principal components for dimensionality reduction to obtain 25 cell clusters after downsampling. (F) Annotation of the 25 clusters using the SingleR R package, categorizing them into 10 cell types after downsampling. Figure R2. Pseudotime analysis of T cells. (A‐C) The developmental trajectory of T cells is inferred by Monocle 3. (D) GZMK expression changes across pseudotime by Monocle 3.

## Data Availability

The data that support the findings of this study are available in the supporting information of this article.
