# Supplementary figures and images for: Single‐Cell Transcriptomics Reveals the Potential Role of GZMK+ CD8+ T Cells in Cell Senescence of Triple‐Negative Breast Cancer
Source: Int J Genomics. 2026 Apr 17;2026:4215646. doi: 10.1155/ijog/4215646 (PMC13088264; doi:10.1155/ijog/4215646)

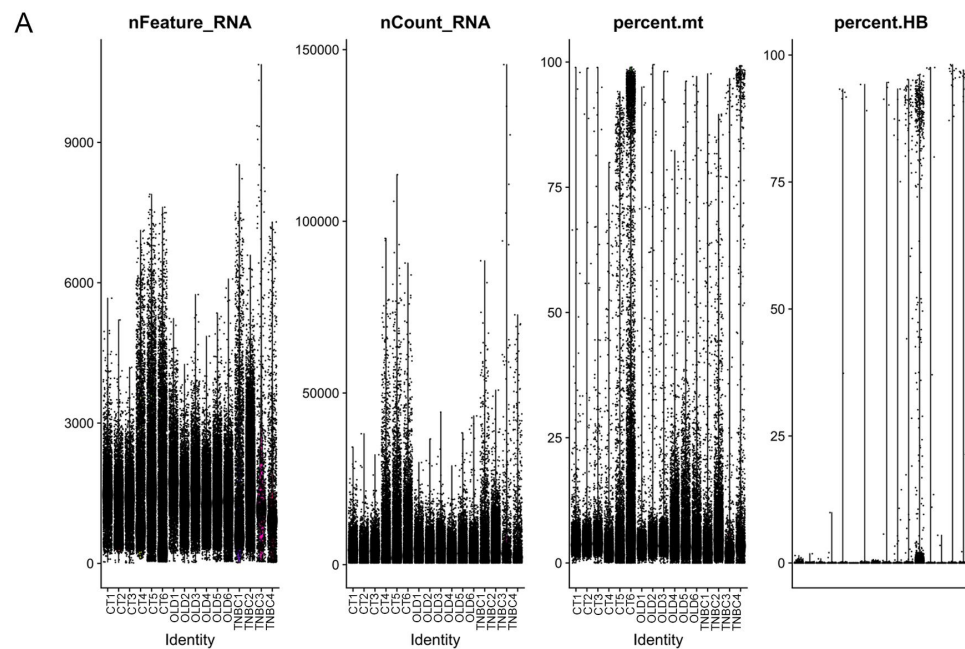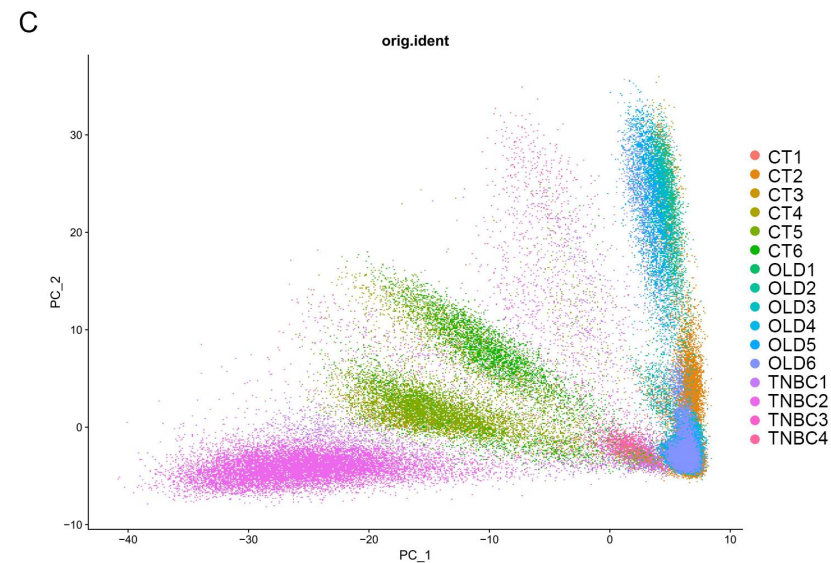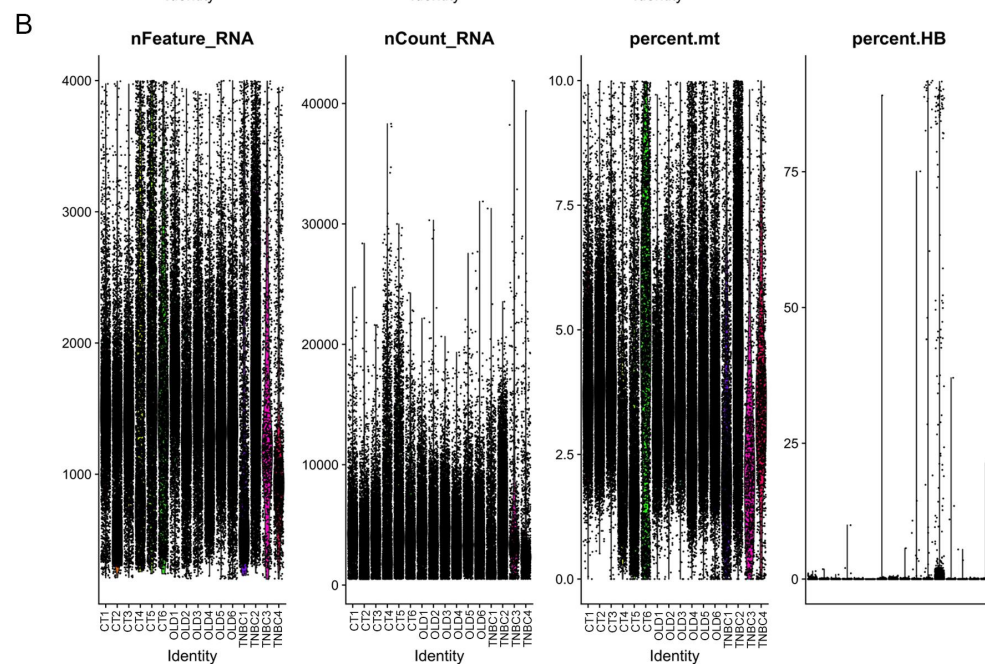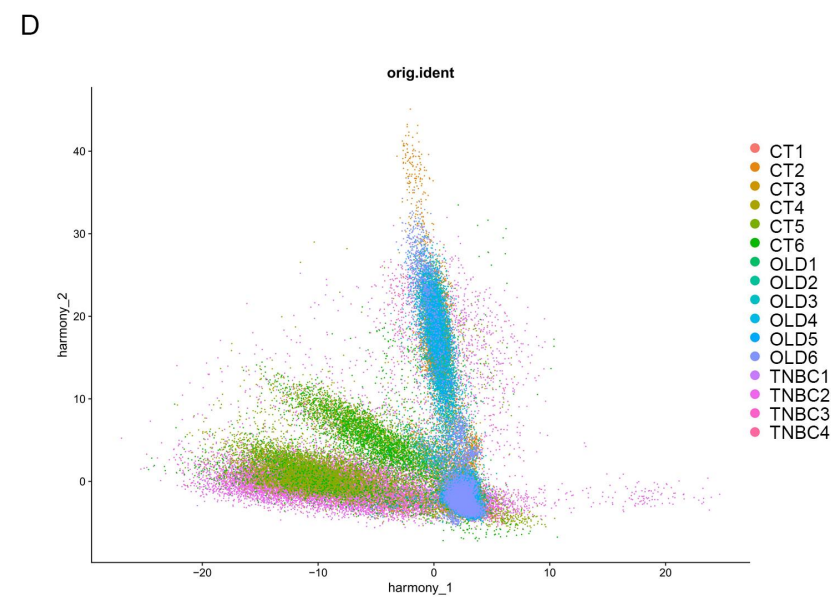

Supplement: Supplementary file 1 — Supporting Information 1 Supporting Information 1: Figure S1. Quality control (QC) of the scRNA‐seq data. Figure S2. The correlation between metabolic pathway and GZMK expression level by gene set enrichment analysis (GSEA) based on TCGA–TNBC cohort. [file IJOG-2026-4215646-s002.zip › figureS1.pdf]

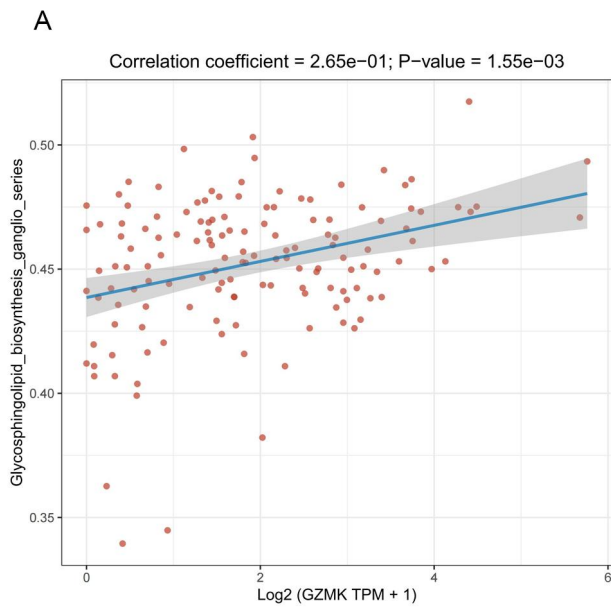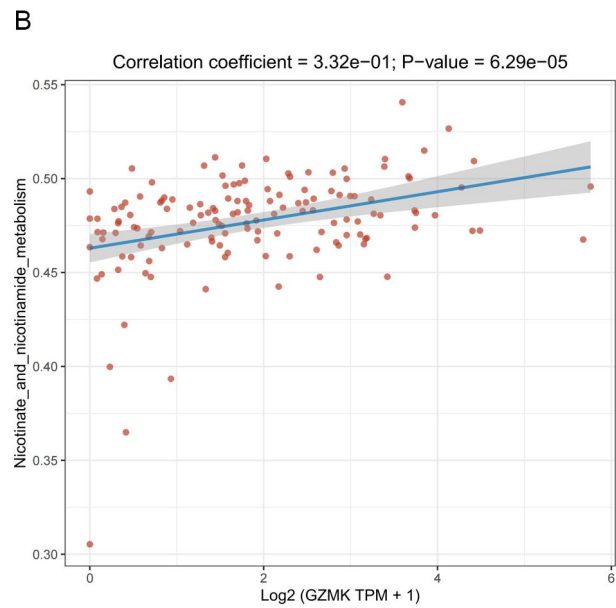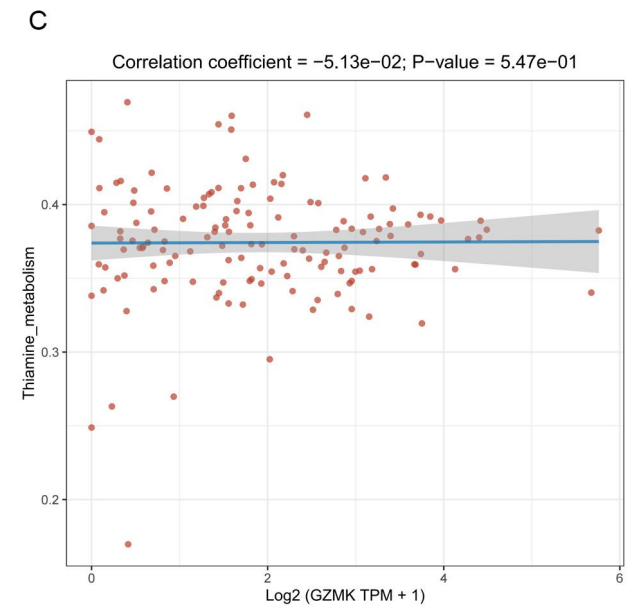

Supplement: Supplementary file 1 — Supporting Information 1 Supporting Information 1: Figure S1. Quality control (QC) of the scRNA‐seq data. Figure S2. The correlation between metabolic pathway and GZMK expression level by gene set enrichment analysis (GSEA) based on TCGA–TNBC cohort. [file IJOG-2026-4215646-s002.zip › figureS2.pdf]

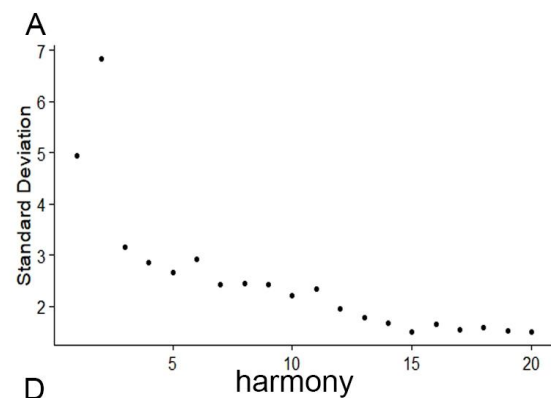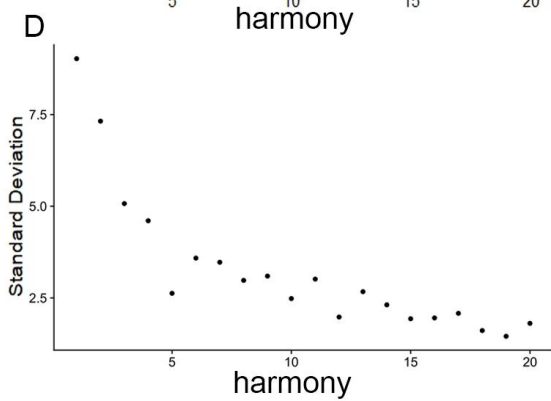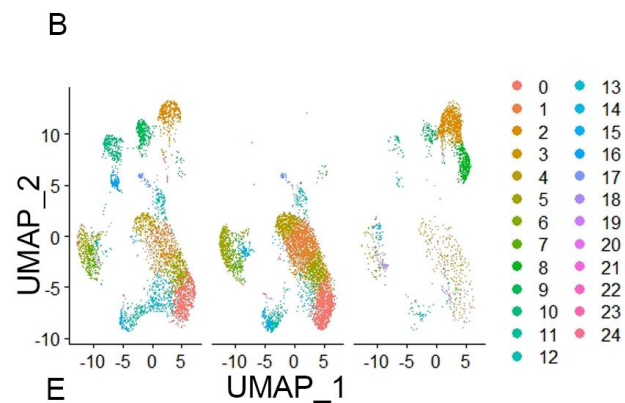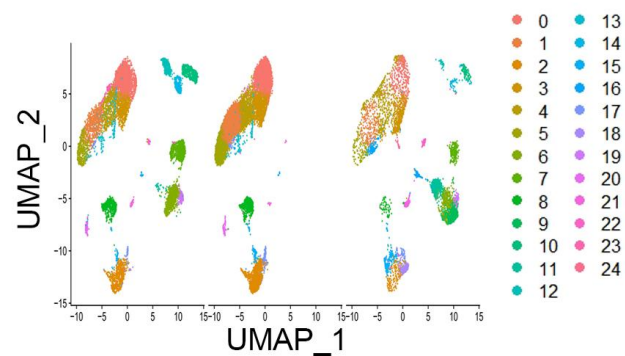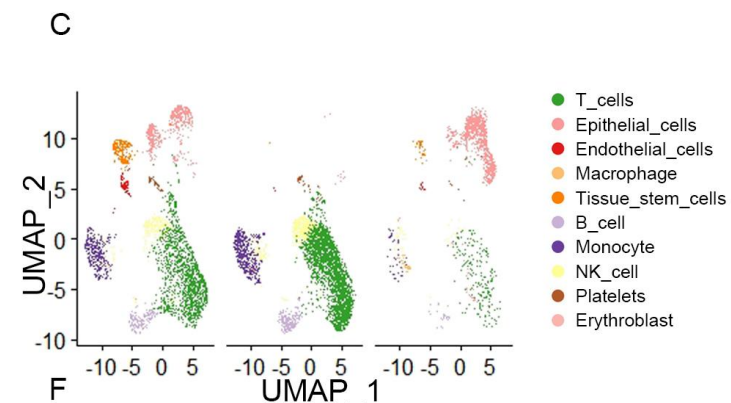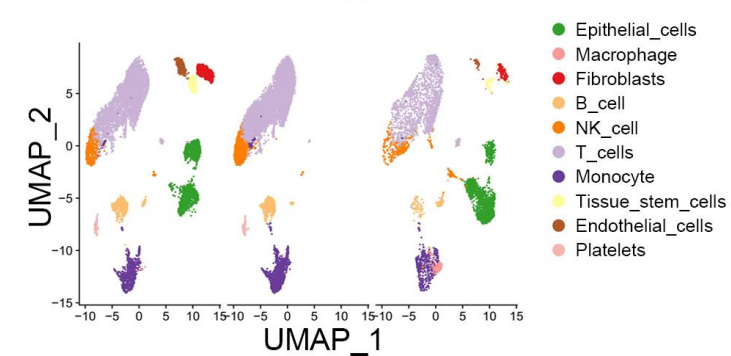

Supplement: Supplementary file 3 — Supporting Information 3 Supporting Information 3: Supporting Data 1. Marker genes of different cell types. Supporting Data 2. Marker genes of different cell types after downsampling. Figure R1. Single‐cell RNA sequencing analysis of breast cancer tissue before and after downsampling. (A) Elbow plots were used to determine the number of principal components for subsequent analyses. (B) The UMAP algorithm was applied to the first 15 principal components for dimensionality reduction to obtain 25 cell clusters. (C) Annotation of the 25 clusters using the SingleR R package, categorizing them into 10 cell types. (D) Elbow plots were used to determine the number of principal components for subsequent analyses after downsampling. (E) The UMAP algorithm was applied to the first 15 principal components for dimensionality reduction to obtain 25 cell clusters after downsampling. (F) Annotation of the 25 clusters using the SingleR R package, categorizing them into 10 cell types after downsampling. Figure R2. Pseudotime analysis of T cells. (A‐C) The developmental trajectory of T cells is inferred by Monocle 3. (D) GZMK expression changes across pseudotime by Monocle 3. [file IJOG-2026-4215646-s001.zip › figureR1.pdf]

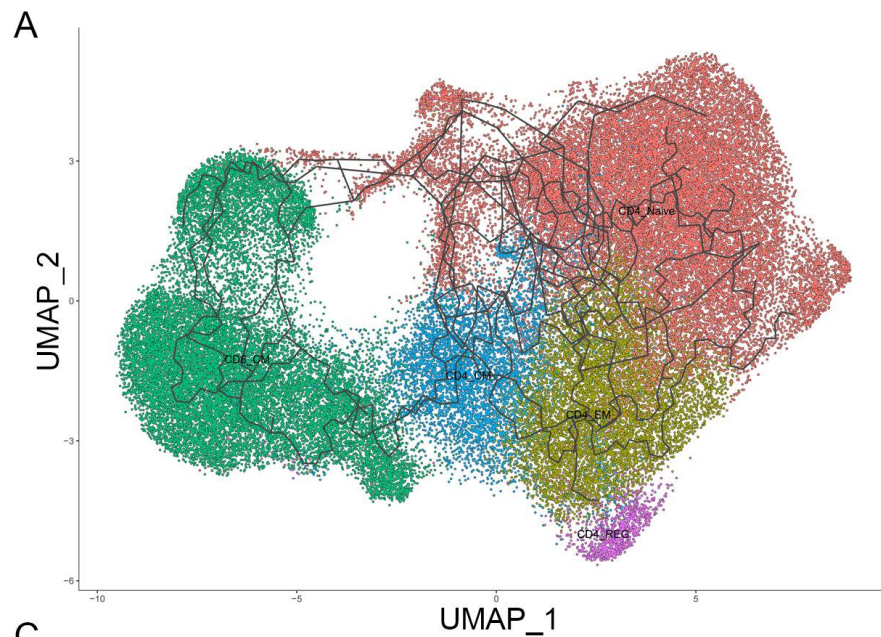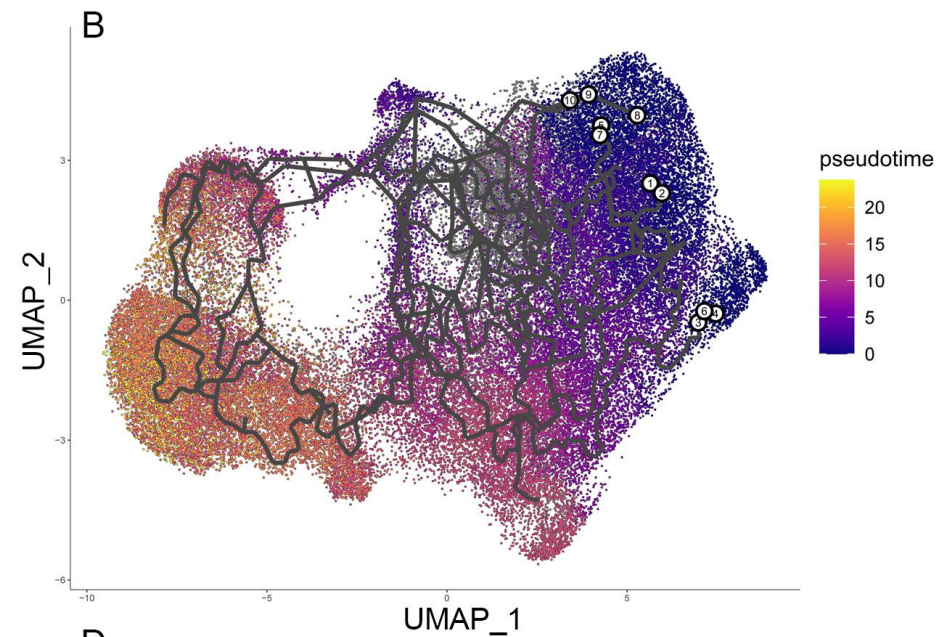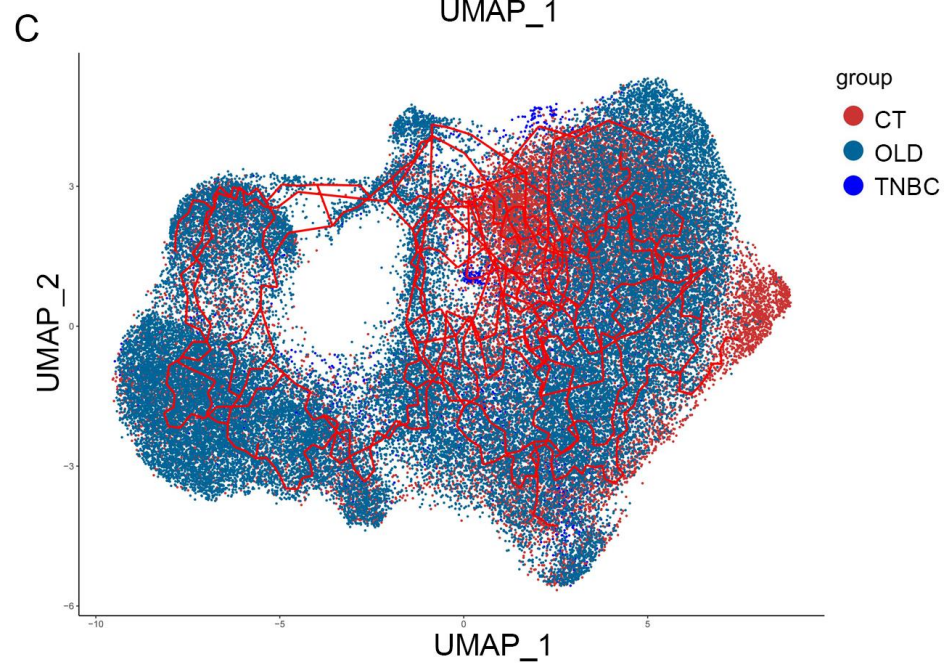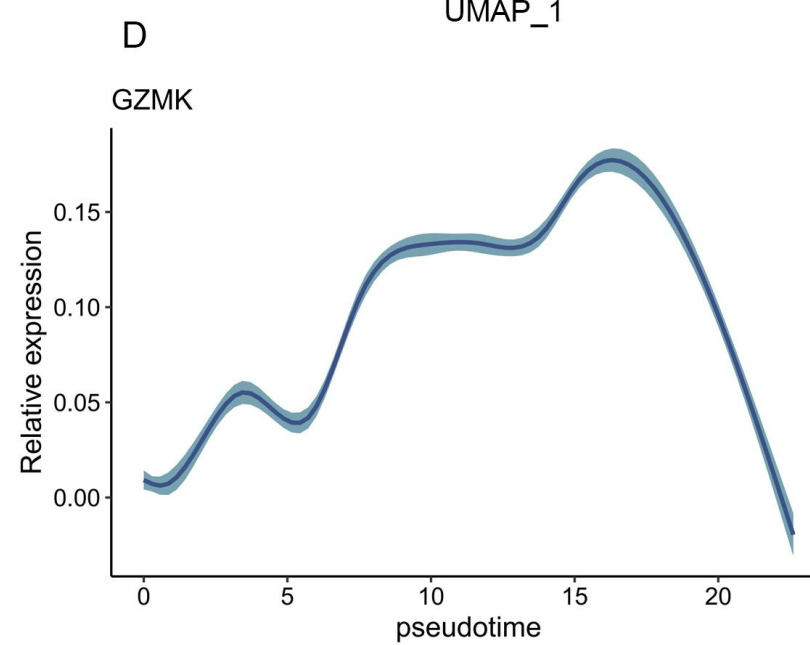

Supplement: Supplementary file 3 — Supporting Information 3 Supporting Information 3: Supporting Data 1. Marker genes of different cell types. Supporting Data 2. Marker genes of different cell types after downsampling. Figure R1. Single‐cell RNA sequencing analysis of breast cancer tissue before and after downsampling. (A) Elbow plots were used to determine the number of principal components for subsequent analyses. (B) The UMAP algorithm was applied to the first 15 principal components for dimensionality reduction to obtain 25 cell clusters. (C) Annotation of the 25 clusters using the SingleR R package, categorizing them into 10 cell types. (D) Elbow plots were used to determine the number of principal components for subsequent analyses after downsampling. (E) The UMAP algorithm was applied to the first 15 principal components for dimensionality reduction to obtain 25 cell clusters after downsampling. (F) Annotation of the 25 clusters using the SingleR R package, categorizing them into 10 cell types after downsampling. Figure R2. Pseudotime analysis of T cells. (A‐C) The developmental trajectory of T cells is inferred by Monocle 3. (D) GZMK expression changes across pseudotime by Monocle 3. [file IJOG-2026-4215646-s001.zip › figureR2.pdf]
